# A sodium-HIF1α axis coordinates immune metabolic reprogramming and mitochondrial remodeling in salt-sensitive hypertension

**DOI:** 10.21203/rs.3.rs-9504540/v1

**Published:** 2026-04-24

**Authors:** Ronald McMillan, Selam Desta, Jeremiah Afolabi, Ariel Thorson, Olivia Pierre-Louis, Ashley Mutchler, Joyanna Gamble-George, Prasanna Katti, Suraj Thapliyal, Mohammad Saleem, Mert Demirci, Lale A. Ertuglu, Sergey Dikalov, Alexandria Porcia Haynes, Andrea Marshall, Mohd khan, Jenny Schafer, Oleg Kovtun, Justin Van Beusecum, Max Kushner, Sharia Yasmin, Cheryl L. Laffer, Thomas R. Kleyman, Celestine Wanjalla, Jeanne A. Ishimwe, Sydney Jamison, Quanhu Sheng, Antentor Hinton, Annet Kirabo

**Affiliations:** 1 Department of Medicine, Division of Genetic Medicine Clinical Pharmacology, Vanderbilt University Medical Center, Nashville, TN, USA; 2 Division of Infectious Diseases, Vanderbilt University Medical Center, Nashville, TN, USA; 3 Department of Molecular Physiology & Biophysics, Vanderbilt University, Nashville, TN, USA; 4 Biomedical Sciences, Meharry Medical College, Nashville, TN, USA; 5 Department of Medicine, Vanderbilt University Medical Center, Nashville, TN, USA; 6 Department of Computer Science, Whiting School of Engineering, Johns Hopkins University, Baltimore, MD; 7 Department of Social and Behavioral Sciences, Yale School of Public Health, New Haven, CT; 8 Department of Epidemiology, Harvard T.H. Chan School of Public Health, Boston, MA; 9 Department of Biology, Indian Institute of Science Education and Research (IISER), Tirupati, AP, India.; 10 Department of Cell and Developmental Biology, Vanderbilt University, Nashville, TN, USA; 11 Department of Chemistry, Vanderbilt University, Nashville, TN, USA; 12 Department of Medicine, Medical University of South Carolina, Charleston, SC, USA; 13 Ralph H. Johnson Veterans Affairs Healthcare System, Charleston, South Carolina, USA.; 14 Renal-Electrolyte Division, Department of Medicine, University of Pittsburgh, Pittsburgh, PA, USA; 15 Veterans Affairs Tennessee Valley Healthcare System, Nashville, Tennessee, USA; 16 Vanderbilt Institute for Infection, Immunology and Inflammation, Vanderbilt University, Nashville, TN, USA; 17 Department of Biostatistics, Vanderbilt University Medical Center, Nashville, TN, USA; 18 Vanderbilt Center for Immunobiology, Vanderbilt University, Nashville, TN, USA; 19 Vanderbilt Institute for Global Health, Vanderbilt University, Nashville, TN, USA

**Keywords:** Hypoxia Inducible Factor 1 Alpha, metabolic reprogramming, antigen-presenting cells, glycolysis, hypertension

## Abstract

Salt-sensitivity of blood pressure (SSBP) is associated with immune-metabolic dysfunction, yet the mechanism that coordinates sodium exposure, mitochondrial remodeling, and blood pressure response remains undefined. Phenome-wide and laboratory-value association studies (PheWAS and LabWAS) in the All of Us Research Program identified fluid, electrolyte, and acid-base balance disorders and renal phenotypes as the strongest disease associations. At the same time, hypertension was linked to reduced serum potassium, chloride, and eGFR, corroborating the centrality of renal-electrolyte physiology in blood pressure regulation. Using a within-subject sodium challenge in humans, we show that sodium loading reorganizes circulating tricarboxylic acid (TCA) cycle intermediates in proportion to the individual blood pressure response. Transcriptomic profiling of immune cells under high sodium revealed suppression of oxidative phosphorylation, induction of HIF1α-dependent glycolytic gene networks, and rebalancing of the pyruvate dehydrogenase complex. Single-cell chromatin accessibility profiling demonstrated that HIF1α motif activity in circulating immune cells correlates with changes in systolic blood pressure and pulse pressure in salt-sensitive individuals. High sodium induced mitochondrial fragmentation with increased organelle mass and glycolytic capacity. Pharmacological HIF1α inhibition reversed fragmentation while only partially normalizing metabolic output, indicating structural and metabolic remodeling are partially dissociable downstream of HIF1α. Renal HIF1α gain-of-function in mice recapitulated the glycolytic transcriptional response with medullary specificity. Concordantly, Drosophila melanogaster subjected to a high-salt diet exhibited impaired locomotor performance, mitochondrial dysmorphology, intestinal barrier disruption, and cardiac remodeling, establishing evolutionary conservation of sodium-induced end-organ dysfunction independent of an adaptive immune system. Together, these findings identify a HIF1α-dependent axis of mitochondrial metabolic adaptation providing a mechanistic basis for SSBP.

## Graphical Abstract

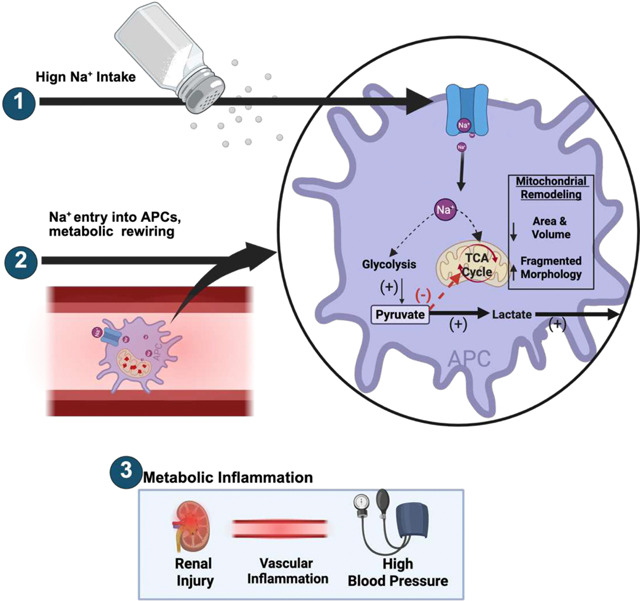

## Introduction

Salt-sensitivity of blood pressure (SSBP) describes a condition in which blood pressure varies with sodium intake, increasing the risk of cardiovascular diseases related to excess sodium intake.^1,2^ SSBP affects an estimated 50% of hypertensive and 25% of normotensive individuals, yet the precise mechanisms underlying its development remain poorly understood.^3^ Salt-sensitive (SS) individuals fail to mount the normal vasodilatory response to sodium loading seen in salt-resistant (SR) individuals, resulting in sustained blood pressure elevation and greater risk for end-organ damage.^4^ Understanding what drives this divergence requires examining how sodium is sensed and processed at the cellular level.

Antigen-presenting cells (APCs) play an important role in the development of SSBP through sodium-driven immune activation.^5^ APCs migrate to high-sodium environments in the vasculature, where sodium entry via the amiloride-sensitive epithelial sodium channel (ENaC) activates NADPH oxidase, generating reactive oxygen species (ROS) and promoting isolevuglandin accumulation.^5^ These events drive cytokine production, T-cell activation, and sustained vascular inflammation.^6^ Sustaining this level of immune activation is energetically demanding. It has been linked to a shift toward aerobic glycolysis in myeloid cells, a state in which cells reduce their reliance on mitochondrial oxidative metabolism and instead prioritize glycolytic energy production.^7^ However, whether this metabolic shift in SSBP represents a coordinated, sodium-specific adaptation rather than a nonspecific inflammatory response remains to be established.

One consequence of immune metabolic reprogramming is the release of tricarboxylic acid (TCA) cycle intermediates into the circulation, where they can exert effects on blood pressure beyond the immune cell itself.^8^ Succinate and fumarate, in particular, are released from metabolically active immune cells and act on vascular and renal targets to promote vasoconstriction and renin release through the succinate receptor GPR91.^9,10^ This suggests that circulating TCA intermediates may serve as a mechanistic link between immune metabolic state and blood pressure regulation in SSBP.^8^ Plasma metabolomic profiling during a controlled sodium challenge provides a translational readout to assess whether immune metabolic reprogramming generates systemic signals linked to hemodynamic changes in SSBP.^11^

Mitochondrial architecture is an important determinant of how immune cells generate and allocate energy.^7^ When mitochondria fragment, electron transport chain efficiency declines, ROS output increases, and cells shift toward glycolytic energy production.^12^ This structural-to-metabolic coupling is regulated by a conserved set of proteins, including the mitochondrial contact site and cristae organizing system (MICOS) and the fusion GTPases OPA1 and MFN2, whose disruption shifts the mitochondrial network toward fragmentation and recapitulates the metabolic profile of inflammatory immune activation.^13,14^ Elevated sodium induces oxidative stress and increases cellular energetic demand, suggesting that sodium-driven mitochondrial fragmentation may actively drive glycolytic commitment in immune cells rather than simply reflecting it.^7^ Identifying the transcriptional mechanism that initiates and sustains this structural-metabolic shift is therefore central to understanding immune metabolic adaptation in SSBP.

HIF1α is a strong candidate for coordinating sodium-driven metabolic remodeling and mitochondrial fragmentation in immune cells.^15^ In myeloid cells, HIF1α promotes aerobic glycolysis by inducing pyruvate dehydrogenase kinase 1 (PDK1) and lactate dehydrogenase A (LDHA) while suppressing oxidative phosphorylation gene networks.^16–18^ Although HIF1α is classically stabilized under low oxygen conditions, it can also be activated under normal oxygen levels when TCA cycle flux is disrupted.^19,20^ Specifically, succinate accumulation resulting from HIF1α-mediated suppression of TCA cycle enzymes inhibits prolyl hydroxylase domain enzymes, preventing HIF1α degradation and creating a feedforward loop in which initial activation becomes self-sustaining.^19^ In pathological hypoxia models, HIF1α promotes mitochondrial fragmentation by favoring fission over fusion, often through DRP1 activation and reduced MFN1 and MFN2 expression.^21^ In hypoxia-driven models, HIF1α inhibition decreases Drp1 and increases Mfn2, altering mitochondrial morphology, while DRP1 inhibition can restore mitochondrial integrity; however, the magnitude of rescue is context dependent.^22^ Sodium accumulation activates HIF1α-dependent pathways in macrophages, and osmotic stress can stabilize HIF1α independent of oxygen availability, positioning sodium as a non-canonical activator of this circuit.^15^ Whether this self-amplifying HIF1α axis links sodium-driven metabolic and structural remodeling to salt-sensitive blood pressure remains unknown.

The purpose of this study was to investigate a HIF1α-dependent axis of immune metabolic adaptation in the context of SSBP. We hypothesized that excess sodium activates an HIF1α-centered pathway in immune cells that suppresses oxidative metabolism, promotes glycolysis, and remodels the mitochondrial network, and that this response is linked to the blood pressure phenotype in SS individuals. To test this hypothesis, we used an integrated approach spanning human plasma metabolomics, bulk and single-cell transcriptomics, chromatin accessibility profiling, in vivo murine modeling, and high-resolution mitochondrial imaging. Evidence across these levels of analysis, rather than any single experiment, supports a mechanistic framework in which sodium-driven immune metabolic remodeling reflects a coordinated and hemodynamically relevant adaptation in SSBP.

## Methods

### All of Us Research Program HTN Case-Control PheWAS and LabWAS:

Study Cohort. Participants were selected from the All of Us Research Program Controlled Tier.^23^ Eligible participants were adults aged 18–65 years with available electronic health record data, short-read whole-genome sequencing data, and (male or female) available for regression modeling. Data was accessed through Curated Data Repository version 8 (CDRv8). The analytic covariate file used for the hypertension case-control analyses contained 49,897 participants after covariate derivation. Hypertension cases were defined using an All of Us EHR-based phenotype that incorporated diagnosis-based and measurement-based criteria, including standard and source concepts for essential hypertension, systolic hypertension, diastolic hypertension, secondary hypertension, hypertensive heart disease, hypertensive chronic kidney disease, and hypertensive heart and chronic kidney disease, as well as repeated elevated blood pressure measurements (systolic blood pressure ≥140 mmHg and/or diastolic blood pressure ≥90 mmHg on ≥3 occurrence dates in adults aged ≥18 years); non-hypertensive controls were defined as participants who did not meet any of these criteria. For hypertension cases, the index date was defined as the first date of a qualifying hypertension diagnosis. Exclusion criteria were evaluated relative to the index date using structured electronic health record data. Specifically, participants were excluded for: acute cardiovascular event(s) within the previous 6 months; inability to participate in protocol-directed assessment; current excessive alcohol or illicit drug use; blood pressure below inclusion thresholds after discontinuation of therapy; presence of metal implants such as artificial joints; concomitant diabetes mellitus type I or II; autoimmune disease; recent vaccination; age outside the eligible range; pregnancy; confirmed or suspected renal, renovascular, or endocrine causes of secondary hypertension; treatment with agents known to increase blood pressure (for example, adrenergic agonists for ADHD, SSRIs/SNRIs, chronic decongestant use, or non-steroidal anti-inflammatory drugs); active or ongoing infection, including HIV/AIDS; active or ongoing malignancy other than basal cell carcinoma of the skin; severe psychiatric disorders; conditions expected to substantially alter immune readouts; treatment with systemic glucocorticoids, immunosuppressants, direct immunomodulators, or chemotherapeutic agents; contraindications to high-salt or low-salt diets or ambulatory blood pressure monitoring; and medical contraindications to study food content. Baseline characteristics were ascertained using data available as of the index date and are summarized in Supplementary Table 1.

### Clinical Phenome-Wide Association Study (PheWAS).

We performed a clinical case-control phenome-wide association study in the All of Us analytic cohort.^24^ Hypertension case status was encoded as the independent variable of interest. Covariates were derived using PheTK (v0.1.47), an All of Us-adapted Python framework for phecode-based phenome-wide association testing, and included age at last EHR event, sex at birth, and the first five genetic principal components; participants with missing covariates were excluded to generate a covariate-complete analytic cohort. The initial analytic case-control cohort comprised 49,897 participants, including 4,187 hypertension cases and 45,710 non-hypertensive controls. After covariate derivation and exclusion of participants with missing covariate data, the final covariate-complete analytic cohort included 27,318 participants, including 3,911 hypertension cases and 23,407 non-hypertensive controls. International Classification of Diseases, Ninth or Tenth Revision, Clinical Modification (ICD-9-CM/ICD-10-CM) diagnosis data were restricted to this cohort and mapped to phecodes using phecode version 1.2 (U.S. ICD mapping). Logistic regression was then performed for each phecode with hypertension case status as the independent variable of interest and phecode case status as the dependent variable, adjusting for age at last EHR event, sex at birth, and the first five principal components. Analyses excluded phecodes with fewer than 50 cases or fewer than 50 controls. In the final clinical PheWAS, 474 phecodes were tested, and statistical significance was assessed using Bonferroni correction based on the number of phecodes tested.^24^

### Clinical Lab-Wide Association Scan (LabWAS).

We performed a clinical laboratory-wide association scan using the covariate-complete hypertension case-control cohort.^25^ Laboratory measurements were extracted from the All of Us measurement table and harmonized across related measurement concepts by cleaning concept names into shared laboratory labels, assigning each label to a laboratory group, and retaining the dominant unit for each harmonized laboratory phenotype. Repeated measurements were summarized at the participant level by taking the median value for each participant within each laboratory phenotype. Extreme values were excluded using a within-laboratory outlier filter (>4 standard deviations from the laboratory mean). Laboratory phenotypes were eligible for testing if they were available in at least 100 participants overall and in at least 20 cases of hypertension. For each retained laboratory phenotype, values were rank-based inverse normal transformed and analyzed using linear regression with hypertension case status as the independent variable of interest, adjusting for age at last EHR event, sex at birth, and the first five genetic principal components. In the final clinical LabWAS, 228 laboratory phenotypes were tested in 27,269 participants, including 3,907 hypertension cases and 23,362 non-hypertensive controls. Target electrolyte and renal laboratory phenotypes were additionally flagged a priori for annotation.

### Human Studies:

Two independent cohorts were recruited and studied. A cohort of 30 hypertensive individuals was rigorously phenotyped for in vivo salt-sensitivity of blood pressure using a modified Weinberger protocol of rapid salt loading and salt depletion (demographic information and clinical data are provided in Table 1 and previously published).^26^ Blood and urine samples were collected from SSBP-phenotyped patients and subsequently studied. Written consent was obtained from all participants prior to enrollment. A separate cohort of 11 healthy individuals was used to perform bulk RNA transcriptomic analysis of isolated monocytes following in vitro high-sodium treatment (demographic information and clinical data are presented in Table 2 and previously published).^27^ All studies were approved by the Institutional Review Board of Vanderbilt University Medical Center, and all procedures were performed in accordance with the Declaration of Helsinki.

### Study Population:

The cohort of SSBP-phenotyped hypertensive individuals used *in vivo* studies included 30 individuals aged 18–65 years with systolic blood pressure >140 mmHg or diastolic blood pressure >90 mmHg. These patients were recruited at Vanderbilt University Medical Center from 2019–2024. Individuals excluded had: (i) history of an acute cardiovascular event within six months before study, (ii) confirmed or suspected renal, renovascular, or endocrine causes of secondary hypertension, (iii) active cancer, (iv) infectious and inflammatory disease (i.e., active infection or connective tissue disorder), (v) treatments that modify immune responses (e.g., immunomodulators, immunosuppressants, glucocorticoids), (vi) treatments that induce elevated blood pressure (e.g., selective serotonin reuptake inhibitors and serotonin and norepinephrine reuptake inhibitors, chronic use of decongestants or non-steroidal anti-inflammatory drugs), and/or (vii) pregnancy. Demographic and clinical data were collected from participants as described previously (Table 1).^28^

The cohort of healthy individuals used for *in vitro* studies comprised 11 women aged 22–46 years with no history of inflammatory diseases. Individuals excluded had: (i) confirmed or suspected renal, renovascular, or endocrine causes of secondary hypertension, (ii) diabetes mellitus, type I or II (iii) treatments requiring corticosteroids or immunosuppressants, (iv) vaccination against an infectious agent within three months before study, (v) active cancer, (vi) severe psychiatric disorders; and (vii) HIV/AIDS. Blood samples were collected, and monocytes were isolated on the same day. Bulk RNA transcriptomic analysis was subsequently performed after 72 hours of high salt treatment. Demographic and clinical data were collected from participants, as previously published (Table 2)^6^.

### Human Study Protocol:

The cohort of hypertensive patients was instructed, prior to the study, to maintain their normal diet and discontinue use of antihypertensive medication for at least a weeks. Salt sensitivity in patients was assessed using a rapid, rigorous salt-loading and salt-depletion protocol at the Vanderbilt University Medical Center Clinical Research Center, as previously described^29^. Blood pressure was measured using an ambulatory blood pressure monitor (Spacelabs 90207). Baseline blood samples were drawn at 8 AM on the first day of the study, followed by a salt-loading diet containing 160 mEq NaCl (prepared by the University of Alabama Bionutrition Core of the Clinical Research Unit Metabolic Kitchen) and a 2 L intravenous infusion of normal saline administered from 8 AM to 12 PM. The following morning, were collected. Salt depletion was achieved by administering three 40-mg doses of oral furosemide at 8 AM, 12 PM, and 4 PM, along with a low-salt diet containing 10 mEq NaCl. The salt depletion blood sample was drawn on the morning of day 3. The participants were discharged following blood collection. Blood pressure and pulse rate were recorded every 15 minutes from 6 AM to 10 PM and every 30 minutes at night on all 3 study days. Baseline blood pressure was recorded as the average blood pressure from 6 AM to 8 AM on day 1. Salt-loading and salt-depletion blood pressures were calculated as the average blood pressure from 12 PM to 10 PM on days 1 and 2, respectively. Blood and urine samples were collected from all participants at baseline, during salt loading, and during salt depletion. Demographic and clinical data were collected from all participants of this cohort, as previously published (Table 1).

### Bulk RNA sequencing:

Heparinized blood (40 mL) was obtained from volunteers, and peripheral blood mononuclear cells were isolated using a Ficoll-gradient protocol. Monocytes were isolated further from the PBMCs by magnetic labeling and negative selection using the Miltenyi monocyte isolation kit (Miltenyi Biotec 130–091-151) and cultured in 12-well plates at 1 × 10^6^/mL density in either normal Na^+^ RPMI media (150 mMol/L Na^+^) or high Na^+^ RPMI media (190 mMol/L Na^+^). Both normal and high Na^+^ media were prepared with RPMI media 1640 (Gibco) containing 10% FBS, 1% pen/strep, and 1% HEPES. Sodium concentrations ranging from 150 to 190 mM were used to simulate both physiological (150 mM) and pathophysiological (190 mM) conditions, as elevated sodium levels are clinically relevant in states such as hypernatremia. This range was selected to assess sodium-sensitive mechanisms and cellular responses under varying extracellular sodium conditions. High-quality RNA was extracted from the samples using RNeasy Midi Kit (Qiagen, Valencia, CA, USA) according to the manufacturer’s protocol. Quality control of the RNA samples was conducted by Vanderbilt Technologies for Advanced Genomics (VANTAGE) core ensuring a high RNA Integrity Number (RIN) value. The Illumina Tru-Seq RNA sample prep kit was used to perform polyadenylated RNA sequencing at the VANTAGE core. Pair-end sequencing was performed on the Illumina HiSeq 2500. Using the R package, the FASTQ data files from the paired-end sequencing analysis were aligned with TopHat 2 for each sample against the human GRCh38 reference genome assembly. Quality control for the RNA-Seq was performed during the following stages: 1) RNA quality; 2) raw read data (FASTQ); 3) alignment; 4) gene expression. Quality control of the raw data and alignment was performed using QC3, and MultiRankSeq was used to analyze expression. False discovery rate (FDR < 0.05) was corrected for multiple hypothesis testing, and comparisons were performed using paired analysis. Expression-based heatmaps were generated using the Heatmapper online tool.^30^

### Cellular Indexing of Transcriptomes and Epitopes by Sequencing (CITE-Seq):

Cell hashing and CITE-Seq analysis were performed as described previously^27^. Isolation of PBMCs was carried out according to the manufacturer’s protocol (Fisher Scientific, Cat# 14–959-51D) in BD Vacutainer^®^ CPT^™^ Mononuclear Cell Preparation tubes. In summary, PBMCs were stained with antibody-oligonucleotide conjugates, and sample-specific hashtags were incorporated to enable sample multiplexing. The complex was fed into the microfluidic system along with custom beads conjugated with poly dT and nucleotide sequences made to capture the hgRNA barcodes. Oil was included to form a small vesicle containing a single bead and a single cell, with an antibody-oligonucleotide complex bound to the bead. The cell was lysed, and intracellular cDNA and Antibody Derived Tags (ADTs) were formed using reverse transcription of the mRNA to cDNA in the VANTAGE facility. 10x Genomics Cell Ranger 6.0.2 was used to quantify genes. In-house scripts were used to demultiplex sample-specific hashtags, and the hashtag abundance cutoff of positive cells was decided by modified R package cutoff. Each cell was classified as singlet with specific hashtag, doublet or negative and genotype-based demultiplex results from Souporcell were integrated with this hashtag-based demultiplex result^31^. Clustering analysis used Seurat with a resolution of 1.0. and the cell type of each cluster was classified based on cell activity database^32,33^. This cell type was manually refined according to cell-specific marker genes. We used edgeR to identify differentially expressed genes across conditions and the WebGestaltR package to perform Genome Ontology and KEGG pathway over-representation analyses on differentially expressed genes.^34,35^ We performed gene enrichment analysis using the GSEA package.^36^

### ATAC Sequencing:

Stored cryopreserved PBMCs were submitted to MedGenome Inc. (Foster City, California, USA) for nuclei isolation and sequencing. PBMCs were thawed, and granulocytes were removed using Miltenyi Biotec CD66abce beads (cat#: 130–092-393). Nuclei were isolated according to the Nuclei Isolation for Single Cell ATAC Sequencing Demonstration Protocol (10X Genomics, CG000169) with RNAse inhibitors to protect RNA quality. ScATAC-seq libraries were created and processed using the Chromium Next GEM Single Cell Multiome ATAC + GEX (v1 chemistry) according to the manufacturer’s protocol (10X Genomics, CG000338 Rev E). Briefly, 5,000 nuclei were mixed with ATAC Buffer and ATAC Enzyme from 10x Genomics to initiate the transposition reaction. Nuclei were partitioned into Gel Beads-in-emulsion (GEMs) Chip J, with each 10X chip gel bead containing a unique barcode for each nucleus to identify poly-adenylated mRNA for gene expression library and transposed DNA fragments for the ATAC library. Preamplification and cleanup of the GEMs were accomplished according to the user guide. The Illumina sequencing handles (P5/P7) and a sample index were added to the preamplified cDNA product using PCR. ATAC and gene expression libraries were sequenced at MedGenome Inc. using Ilumina Novaseq 6000 S4 (200 cycle) and Novaseq 6000 SP (100 cycle), respectively. ScATAC-seq data were aligned and processed using CellRanger-arc-2.0.0. with a human reference transcriptome (hg19). Data were analyzed with the 10X Genomics Loupe Browser. Quality thresholds were set, and cells with less than 500 RNA UMI counts, or 1000 feature counts with greater than 15% mitochondrial UMI counts were excluded from further analysis.

### Metabolomic Profiling:

Metabolomic profiles of plasma and urine samples of nine hypertensive individuals phenotyped for salt-sensitivity were performed at Metabolon, as previously published (Metabolon Inc., Morrisville, NC, USA; global metabolomics platform).^37,38^ Samples were prepared with the automated MicroLab STAR^®^ system from Hamilton Company. For quality control, multiple recovery standards were included before the first step in the extraction process. Proteins were precipitated with methanol under vigorous shaking for 2 min (Glen Mills GenoGrinder 2000) to remove proteins and dissociate small molecules, thereby obtaining chemically diverse metabolites. Centrifugation was subsequently performed and the resulting extract was separated into five groups: one for analysis by HILIC/UPLC-MS/MS with negative ion mode electrospray ionization (ESI), one for analysis by reverse phases (RP)/UPLC-MS/MS with negative ion mode ESI, two for analysis by two separate RP/UPLC-MS/MS methods with positive ion mode ESI, and one withheld for backup. Samples were placed briefly on a TurboVap^®^ (Zymark) to remove the organic solvent. The resulting extracts were stored under nitrogen overnight before analysis. Metabolite detection was performed on a per-sample basis and expressed as peak area (i.e., the integrated area under the curve).

### Nanostring GeoMX digital spatial imaging profiling:

Kidney tissues from wild-type (WT) and high-salt diet (HSD)-fed epithelial sodium channel (ENaC) gain-of-function (GOF) mice were harvested and processed as either formalin-fixed paraffin-embedded (FFPE) or fresh-frozen sections. Tissue sections were mounted on glass slides and subjected to immunofluorescence staining with antibodies against DAPI (nuclear marker), PanCK (epithelial marker), and CD45 (immune cell marker) to provide morphological context for region-of-interest (ROI) selection. Spatial transcriptomic profiling was then conducted using the NanoString GeoMx Digital Spatial Profiler (DSP) platform. Stained slides were loaded onto the GeoMx DSP instrument for whole-slide imaging, utilizing up to four fluorescent channels to visualize tissue architecture and guide ROI selection within the renal cortex and medulla. For gene expression analysis, sections were incubated with a custom panel of oligonucleotide-barcoded probes targeting transcripts associated with glycolysis and HIF1A signaling. After hybridization, UV light was used to photocleave oligonucleotide tags from each ROI, which were then aspirated and collected for downstream quantification. Oligonucleotide tags were quantified using either NanoString nCounter technology or next-generation sequencing according to manufacturer protocols. Raw digital counts were spatially mapped to generate region-specific gene expression profiles. Quality control measures included excluding ROIs with low nuclear counts, inadequate surface area, or poor image alignment, as well as removing genes with low expression in more than 90% of ROIs. Negative control probes were used to define background signal, and raw counts were normalized and transformed into log-counts-per-millionusing the edgeR package. Differential gene expression between WT and GOF groups in both the cortex and medulla was assessed using the limma-voom pipeline with multiple testing correction. Representative immunofluorescence images were captured to demonstrate ROI selection and tissue morphology. At the same time, violin plots and other statistical visualizations were employed to depict normalized gene expression and significance levels across experimental conditions.

### HIF1α Transcription Factor Assay:

Stored PBMCs collected at salt loading (Day 2 of in-patient protocol) and salt depletion (Day 3 of in-patient protocol) underwent nuclear protein isolation using Invitrogen Cell Extraction Buffer (FNN0011), Halt Protease Inhibitor (78430), and phenylmethyl sulfonyl fluoride (PMSF). Nuclear extracts were then analyzed using the commercially available HIF1α Transcription Factor Assay Kit (ab133104) according to the manufacturer’s guidelines.

### Live-Cell Imaging and Analysis:

HeLa cells were cultured at a density of 50,000 cells per dish and allowed to adhere overnight. Following visual confirmation of cell adherence, the cells were subjected to the following treatment conditions: The HIF1A inhibitor (MedChem Express, Catalog No. HY-15836) was used at a concentration of 10 nM. Mitochondrial control treatments were performed at the following concentrations: MFN2 (MFI8, MedChem Express, Catalog No. HY-150031) at 20 μM for 6 hours, OPA1 (MYLS22, Millipore Sigma, Catalog No. AMBH97B9F609) at 50 μM for 48 hours, and Micos (Miclxin, MedChem Express, Catalog No. HY-138301) at 30 μM for 1 hour. Live-cell mitochondrial dynamics were visualized using a Nikon Eclipse Ti2 inverted fluorescence microscope equipped with a Yokogawa CSU-W1 spinning disk confocal scanner, Hamamatsu Fusion BT camera, SoRa super-resolution module, environmental chamber, piezo stage controller, and solid-state lasers (405, 488, 561, and 640 nm), all controlled via NIS-Elements AR software (version 5.42). Cells cultured in 35-mm MatTek glass-bottom dishes were imaged with a 100× Plan Apo Lambda D oil immersion objective (NA 1.45), capturing MitoTracker Orange-labeled mitochondria through the 561 nm channel at 2.5-second intervals over 5 minutes using either standard W1 mode (xy pixel size: 65 nm) or SoRa mode (xy pixel size: 23 nm), with laser power maintained at 5% and 100 ms exposure time per frame to balance phototoxicity concerns with signal quality (SNR ~1.5–2.5). The Perfect Focus System maintained z-stability throughout all acquisitions. At the same time, high-resolution z-stacks (10–20 μm thick) were acquired in SoRa mode with 100 nm step sizes and subsequently enhanced using the Nikon Batch Deconvolution module (version 6.10.02), implementing Blind and Richardson-Lucy algorithms with 20 iterations and automatic noise estimation. SORA imaging and image processing were performed, in part, through the Vanderbilt Cell Imaging Shared Resource and Nikon Center of Excellence.

#### Mitochondrial Volume, Surface Area, and Morphology

Mitochondria were segmented using the Imaris Surface module, which identifies individual mitochondria and interconnected mitochondrial networks as discrete surface objects. Image background subtraction was done at 37.1 μm. Surface generation was performed using a Gaussian smoothing filter with a width of 0.129μm to reduce high-frequency noise prior to segmentation. Segmentation was carried out using the Imaris machine-learning pixel-classification workflow to improve the separation of mitochondrial signal from background. The trained classifier was generated using representative images and applied uniformly across all samples without further adjustment. Objects smaller than 121 voxels were excluded to remove noise and sub-resolution structures. Concentric spatial analysis was performed using Fiji (ImageJ, NIH). Z-stacks were first processed using sum projections to preserve total fluorescence signal across optical sections. A 10 μm diameter region of interest was defined to represent the nuclear zone, and subsequent concentric ROIs were generated in 5 μm radial increments extending outward from the nuclear boundary. Integrated density was measured within each zone to quantify spatial distribution of signal intensity.

### Electron Paramagnetic Resonance (EPR) Spectroscopy:

HeLa cell cultures were collected at the indicated experimental conditions and prepared for EPR analysis according to established protocols. Measurements of glycolytic activity and superoxide production were performed using spin probe–based EPR spectroscopy. All EPR data acquisition and processing were conducted by the Vanderbilt University Medical Center Free Radicals In Medicine Core (FRIMCORE) using standardized instrumentation and analysis workflows.^5^

#### Transmission Electron Microscopy

As previously described (Hinton et al. 2023), cells were fixed in 2.5% glutaraldehyde diluted in sodium cacodylate buffer for 1 h at 37°C and then embedded in 2% agarose, postfixed in buffered 1% osmium tetroxide, stained with 2% uranyl acetate, and dehydrated with a graded ethanol series. Following EMbed-812 resin embedding, 80-nm sections were cut on an ultramicrotome and stained with 2% uranyl acetate and lead citrate. Images were acquired on a JEOL JEM-1230 transmission electron microscope operating at 120 kV.

NIH ImageJ software (Schneider et al. 2012) was used to manually trace and analyze all mitochondria or cristae using the freehand tool (Parra et al. 2013). Measurements of mitochondrial area, circularity, and number were performed using the Multi-Measure ROI tool in ImageJ (Lam et al. 2021; Neikirk, Vue, et al. 2023; Parra et al. 2013). We used three distinct ROIs, all at the same magnification, in ImageJ to examine cristae morphology and determine their area and number. The sum of the total cristae area divided by the total mitochondrial area was used as a proxy to determine cristae volume (Patra et al. 2016).^39–41^

### Locomotor and Flight Performance Assays:

Locomotor and flight performance were evaluated in adult Drosophila melanogaster under the indicated experimental conditions. To assess climbing ability, cohorts of 15–20 age- and sex-matched flies were introduced into a vertical graduated vial and permitted to acclimate prior to testing. Following gentle mechanical displacement to the base of the vial, the proportion of flies ascending to a height of 8 cm within 10 seconds was recorded. A minimum of three independent biological replicates, each comprising multiple technical replicates, were performed per condition. Results were expressed as the percentage of flies reaching the defined threshold height.

Flight capacity was evaluated using a vertical column drop assay, in which individual flies were released from the apex of a cylinder with an adhesive inner surface. Landing position along the column was used to classify flies as strong fliers, weak fliers, or non-fliers. A minimum of 30–50 flies per condition were assessed across independent biological replicates, and flight performance was reported as either a flight index or the percentage of flight-capable individuals. All data are expressed as mean ± standard error of the mean (SEM). Between-group differences were evaluated using unpaired two-tailed Student’s t-tests for pairwise comparisons, or one-way ANOVA followed by appropriate post hoc testing for multi-group analyses. A p-value of less than 0.05 was considered statistically significant.

### Statistical analysis:

Pearson correlation coefficient was used to measure the linear correlation between variables. Statistical significance was set to a p-value of 0.05. Trendlines were estimated using linear regression. The edgeR package was used to identify differential gene expression between conditions with an absolute fold change greater than 1.5 and an FDR-adjusted p-value less than 0.05. GraphPad Prism Version 10.0.2 was used for all statistical analyses. Expression-based heatmaps were prepared using the Heatmapper online tool.

## Results

### Clinical hypertension case-control PheWAS and LabWAS in All of Us support salt-, electrolyte-, and kidney-related phenotypes.

**Figure 1.**
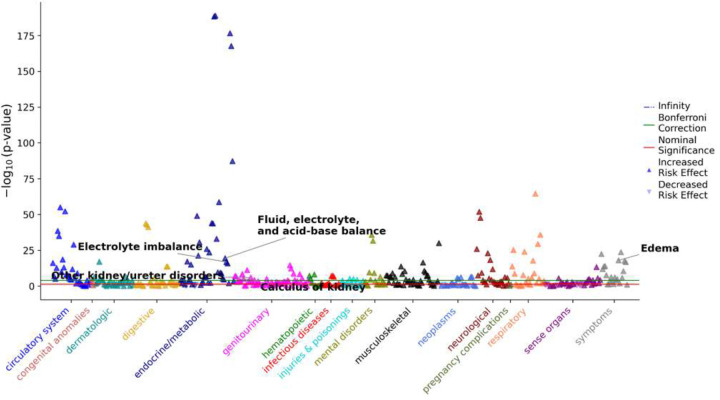
Clinical PheWAS of hypertension versus non-hypertensive controls in the All of Us Research Program. Each point represents a single phecode tested in covariate-adjusted logistic regression, with hypertension case status modeled as the independent variable of interest and phecode case status as the outcome. Phecodes were derived from ICD-9-CM and ICD-10-CM diagnoses mapped using phecode version 1.2 and arranged by clinical category on the x-axis. The y-axis shows -log10(p-value). Upward triangles denote phecodes enriched in hypertension cases, and downward triangles denote phecodes depleted in hypertension cases. Horizontal lines indicate the nominal significance and Bonferroni correction thresholds. Selected salt-, electrolyte-, edema-, and kidney-related phenotypes are annotated.

**Figure 2.**
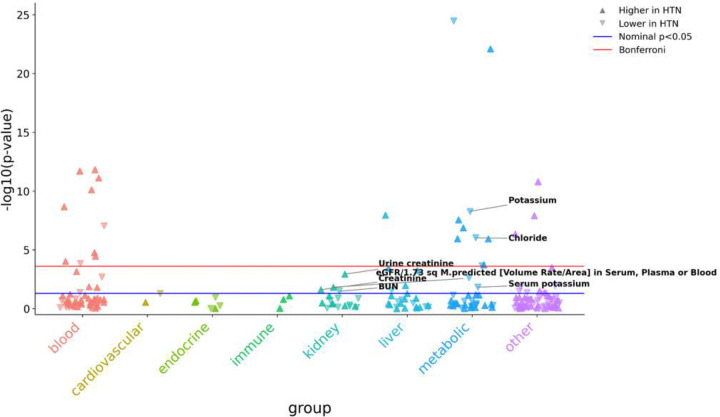
Targeted clinical LabWAS showing electrolyte-, metabolic-, and renal-related laboratory phenotypes associated with hypertension in the All of Us cohort. Each point represents a laboratory phenotype tested in the clinical hypertension-versus-control LabWAS in the All of Us Research Program. Laboratory phenotypes are grouped by domain on the x-axis and plotted by -log10(p-value) on the y-axis. Upward triangles indicate laboratory phenotypes increased in participants with hypertension, whereas downward triangles indicate laboratory phenotypes decreased in participants with hypertension. The blue horizontal line denotes nominal significance (p < 0.05), and the red horizontal line denotes the Bonferroni-correction threshold. Selected electrolyte-, metabolic-, and kidney-related laboratory phenotypes prioritized for interpretation are annotated, including potassium, chloride, serum potassium, urine creatinine, creatinine, blood urea nitrogen (BUN), and estimated glomerular filtration rate (eGFR). Models were adjusted for age at last EHR event, sex at birth, and the first five genetic principal components.

To place our mechanistic findings in a clinical context, we performed a hypertension case-control PheWAS (Figure 1) and LabWAS (Figure 2) in the All of Us Research Program. This orthogonal analysis was designed to test whether hypertension in a large EHR-linked population is enriched for diagnoses and laboratory phenotypes that overlap with the sodium-, electrolyte-, and kidney-related pathways implicated by our metabolomic and immunometabolic data. The analytic covariate cohort for the clinical case-control analysis contained 49,897 participants after covariate derivation. In the clinical PheWAS, hypertension was strongly associated with salt- and kidney-relevant phecodes, including disorders of fluid, electrolyte, and acid-base balance (OR = 4.91, p = 3.01 × 10–20), edema (OR = 4.53, p = 9.78 × 10–19), electrolyte imbalance (OR = 5.08, p = 1.52 × 10–17), other disorders of the kidney and ureters (OR = 3.19, p = 8.90 × 10–8), and calculus of kidney (OR = 2.35, p = 8.51 × 10–6). These associations are directionally consistent with the physiological framework of sodium-sensitive and cardiorenal dysregulation suggested in the mechanistic sections of this study.

In the clinical LabWAS, the full scan was dominated by blood-pressure and anthropometric measurements, as expected for a hypertension case-control comparison. We therefore focused interpretation on clinically relevant metabolic, electrolyte, and renal phenotypes. Among the nominally significant laboratory associations, hypertension was associated with lower HDL cholesterol (p = 3.46 × 10–25), higher triglycerides (p = 8.07 × 10–23), lower potassium (p = 5.34 × 10–9), lower chloride (p = 9.43 × 10–7), higher urine creatinine (p = 1.15 × 10–3), higher estimated glomerular filtration rate (p = 1.48 × 10–2), higher serum creatinine (p = 2.44 × 10–2), and lower urea nitrogen (p = 3.36 × 10–2). These laboratory findings provide clinical context linking hypertension with electrolyte, metabolic, and renal phenotypes that overlap the sodium-responsive pathways suggested by our human dietary-intervention and in vitro datasets.

### Acute Salt Loading/Depletion Alters TCA Cycle Metabolites and Reveals Metabolic Signatures Linked to Salt-Sensitivity of Blood Pressure

**Figure 3.**
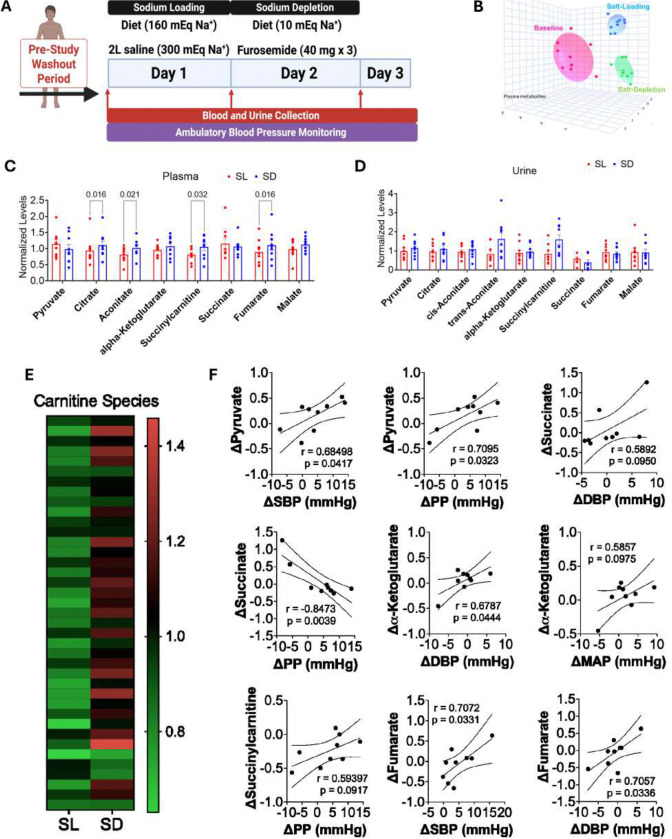
Excess sodium shifts the metabolome in salt-sensitive hypertension. (A) Schematic of the within-subject dietary sodium challenge protocol. (B) Three-dimensional principal component analysis of plasma metabolite profiles at baseline, salt-loading (SL), and salt-depletion (SD). (C) Normalized plasma concentrations of tricarboxylic acid (TCA) cycle intermediates at salt-loading (SL, red) and salt-depletion (SD, blue). (D) Normalized urinary concentrations of TCA cycle intermediates at salt-loading and salt-depletion. (E) Heatmap of acylcarnitine species at SL and SD. Bright green, black, and bright red represent the lowest, median, and highest values, respectively. Rows represent individual carnitine derivatives, while columns represent group averages for the indicated condition. (F) Scatter plots illustrate the relationship between within-subject changes (Δ) in TCA metabolites and blood pressure parameters across the sodium challenge. Δ values represent salt-loading minus salt-depletion. Statistical comparisons were performed using linear regression and Pearson correlation. Pearson r and p values are shown for each comparison. SBP, systolic blood pressure; DBP, diastolic blood pressure; PP, pulse pressure; MAP, mean arterial pressure. Data are presented as mean ± SEM.

To investigate whether excess sodium alters tricarboxylic acid (TCA) cycle metabolism, we conducted metabolomic profiling of plasma and urine samples from nine individuals undergoing an established in-patient salt-loading/salt-depletion protocol. Samples were collected at three time points: baseline (pre-visit), salt loading (Day 1), and salt depletion (Day 2). On Day 3, the participants were monitored and discharged following the study protocol. Participant demographics are summarized in Table 1. Detailed clinical characteristics and sodium excretion data are provided in Figure S1. Salt loading induced a broad shift in the circulating and urinary metabolomes in both SS and SR individuals (Figure 3B). Specifically, salt-loading significantly decreased plasma levels of key TCA cycle intermediates, including citrate, aconitate, succinyl carnitine, and fumarate (Figure 3C). In urine, we did not observe similar trends (Figure 3D). These reductions suggest impaired TCA cycle flux or increased metabolic consumption during salt-induced stress. Because carnitine derivatives play a key role in energy metabolism, we further analyzed their response to the protocol. Salt-loading lowered circulating carnitine-derived species, while these levels rebounded during salt-depletion (Figure 3E), indicating dynamic regulation of energy substrate availability. We next examined relationships between metabolite changes and BP. Changes in systolic blood pressure (SBP) and pulse pressure (PP) from salt-loading to salt-depletion paralleled changes in plasma pyruvate. Additionally, PP changes were negatively correlated with plasma succinate, whereas diastolic BP (DBP) changes were positively correlated with succinate (Figure 3F). Changes in plasma α-ketoglutarate positively correlated with DBP and mean arterial pressure (MAP), while fumarate levels tracked with both SBP and DBP. Notably, plasma succinyl carnitine positively correlated with changes in PP.

### High sodium exposure causes differential expression of glycolytic genes in human monocytes *in vitro*

**Figure 4.**
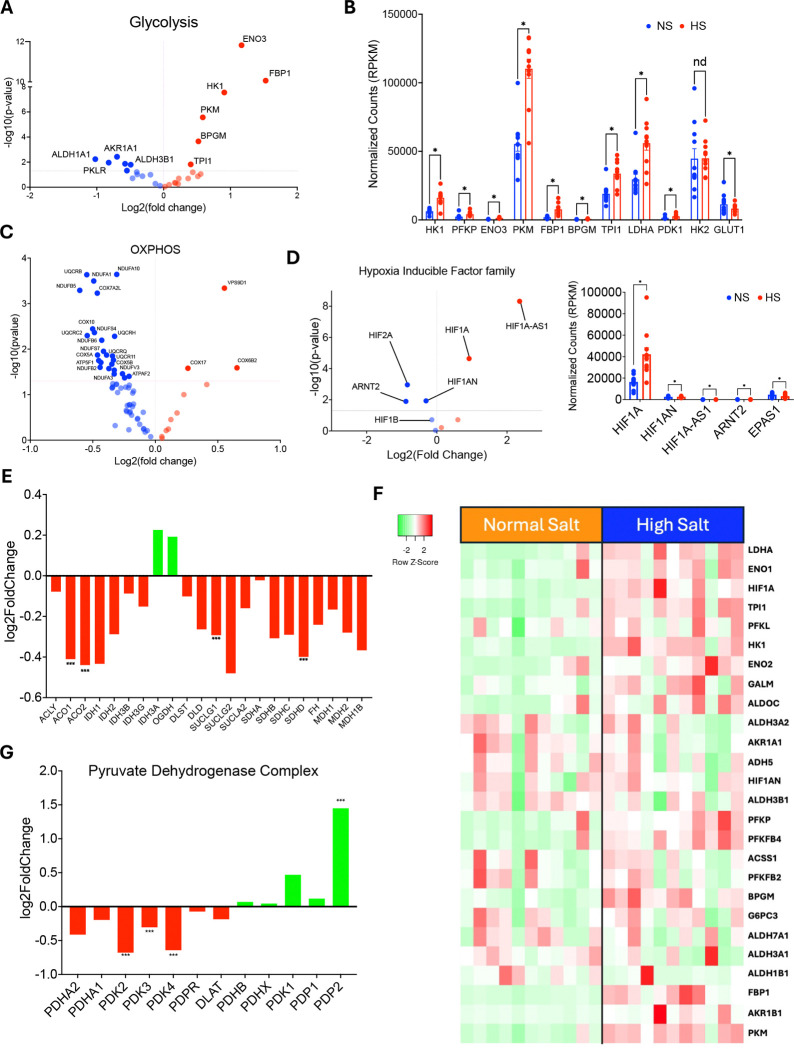
Excess sodium upregulates glycolytic genes in human monocytes *in vitro* (A) Volcano plot illustrating differentially expressed glycolytic genes measured during bulk RNA sequencing analysis. (B) Reads per kilobase per million mapped reads (RPKM) for glycolytic genes in normal sodium (150 mM Na^+^) or excess sodium (190 mM Na^+^) environments. (C) Volcano plot illustrating differentially expressed genes involved in oxidative phosphorylation. (D) Volcano plot demonstrating differentially expressed members of the Hypoxia Inducible Factor (HIF) family and RPKM for HIF family genes. (E) Bar charts demonstrating differential expression from normal sodium to high sodium of genes involved in the tricarboxylic acid (TCA) cycle. (F) Heat map visualizing differential expression of glycolytic genes. Bright green, white, and bright red represent the lowest, median, and highest expression levels, respectively. Rows represent individual genes, while columns represent individual samples (N=11). (G) Differential expression of the gene members of the pyruvate dehydrogenase complex. Statistical comparisons between normal salt (NS) and high salt (HS) conditions were performed using unpaired two-tailed Student’s *t*-tests. Significance thresholds are indicated in each panel, with *p* values (* = p >.05) shown where applicable. Data are presented as mean ± SEM.

Previously, we have shown that increased extracellular sodium induces a DC-like phenotype in human monocytes, leading to increased T cell activation^42^. To investigate whether sodium upregulates glycolysis-related genes in monocytes to promote migratory behavior, we isolated monocytes from healthy individuals (n = 11), exposed them to either normal (150 mMol/L) or high (190 mMol/L) sodium for 72 hours *in vitro*, and subsequently performed bulk RNA sequencing. The demographic information of these participants is presented in Table 2. Transcriptomic analysis revealed differential expression of glycolytic genes (Figure 4A). We found that high sodium levels increase LDHA (lactate dehydrogenase A) expression, a gene regulated by the transcription factor HIF1α (Figure 4B). HK2 (hexokinase 2) and PDK1, also known targets of HIF1α, showed increased expression, but these changes were not statistically significant (Figure 4B). Notably, high sodium significantly upregulated several glycolysis-related genes, including HK1, PFKP, ENO3, PKM, FBP1, BPGM, and TPI1 (Figure 4B). At the same time, high sodium reduced the expression of many genes involved in oxidative phosphorylation (Figure 4C, see also Figure S5 for an enlarged version). When we examined HIF family genes, they were significantly upregulated in monocytes exposed to high sodium (Figure 4D). In addition, high sodium significantly decreased the expression of several genes involved in the TCA cycle, such as ACO1, ACO2, SUCLG1, and SDHD (Figure 4E). These findings were consistent across gene expression data from normal and high-salt-treated immune cells (Figure 4F). We also examined genes in the pyruvate dehydrogenase complex and observed that PDK2, PDK3, and PDK4 were significantly downregulated under high sodium, whereas PDK1 and PDP2 were upregulated (Figure 4G).

### Salt-loading/depletion causes fluctuations in HIF1α motif activity in salt-sensitive but not salt-resistant subjects

**Figure 5.**
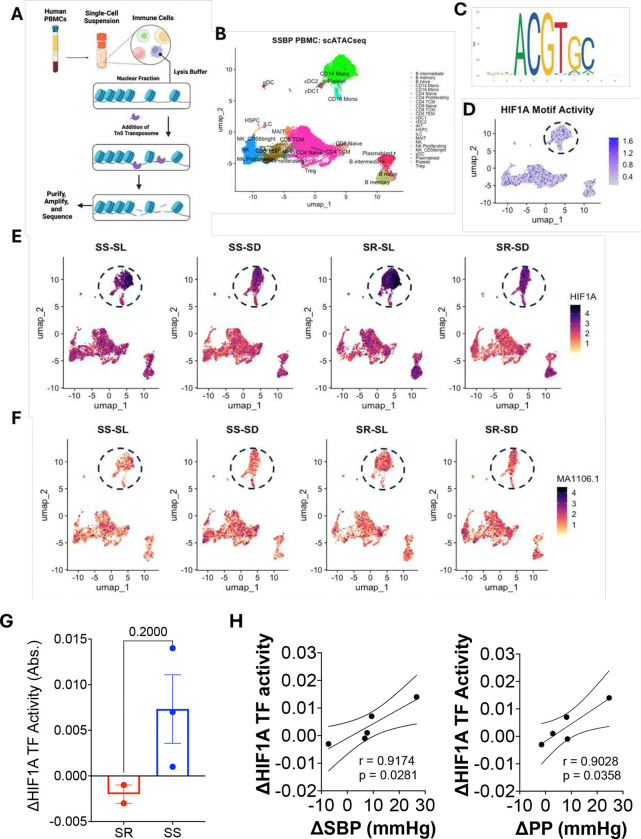
HIF1A transcriptional activity positively correlates with changes in blood pressure *in vivo* (A) Schematic illustration of protocol for Assay for Transposase-Accessible Chromatin (ATAC) with high-throughput sequencing using human Peripheral Blood Mononuclear Cells (PBMCs). (B) UMAP representation of different immune cell clusters identified with antibody-derived tags. (C) Schematic illustration of the HIF1A binding motif used to analyze differential chromatin accessibility. (D) UMAP illustration demonstrating HIF1A binding motif accessibility in different immune cell clusters in a select salt-sensitive patient. (E) UMAP representation of HIF1A gene expression at salt-loading and salt-depletion in select salt-sensitive and salt-resistant patients. (F) UMAP representation of HIF1A binding motif accessibility at salt-loading and salt-depletion in select salt-sensitive and salt-resistant patients. (G) Changes in HIF1A transcriptional activity from salt-depletion to salt-loading in isolated MHC Class II presenting cells in select salt-sensitive and salt-resistant patients. (H) Scatter plots demonstrating correlations between changes in blood pressure and changes in HIF1A transcriptional activity from salt-depletion to salt-loading.

To determine whether HIF1α contributes to salt sensitivity through changes in chromatin accessibility rather than transcriptional abundance, we performed single-cell ATAC-seq on PBMCs from SS and SR individuals under salt-loading and salt-depletion conditions (Figure. 5A). Immune cell populations were resolved by UMAP, revealing canonical clustering across major immune subsets, including myeloid and lymphoid compartments (Figure. 5B). Analysis of transcription factor motif enrichment identified accessibility of the HIF1α binding motif (ACGTGC) across immune cell clusters (Figure. 5C). Projection of HIF1α motif activity onto the UMAP revealed localized enrichment within a subset of immune cells, most prominently within the circled cluster (Figure. 5D). Stratification by salt sensitivity and dietary sodium status demonstrated dynamic changes in HIF1α motif activity in SS individuals between salt-loading and salt-depletion, whereas SR individuals exhibited stable motif accessibility across conditions (Figure. 5E). In contrast, accessibility of the MA1106.1 motif did not show comparable salt-dependent fluctuation across SS or SR conditions, indicating specificity of the observed chromatin changes to HIF1α-related regulatory elements (Figure. 5F). MA1106.1 corresponds to the HIF1 (ARNT) binding motif, which is constitutively active and was included as a specificity control to distinguish HIF1α-dependent chromatin remodeling from global changes in HIF complex accessibility.^43^ Quantification of changes in HIF1α transcription factor activity revealed a trend toward increased ΔHIF1α activity in SS individuals compared with SR individuals, although this difference did not reach statistical significance (Figure. 5G). Notably, changes in HIF1α motif activity were strongly correlated with changes in systolic blood pressure (ΔSBP) and pulse pressure (ΔPP) across individuals, with greater increases in blood pressure associated with greater increases in HIF1α transcription factor activity (Figure). 5H).

### Renal leukocyte infiltrates in HSD-fed ENaC gain-of-function mice exhibit elevated expression of glycolytic genes

**Figure 6.**
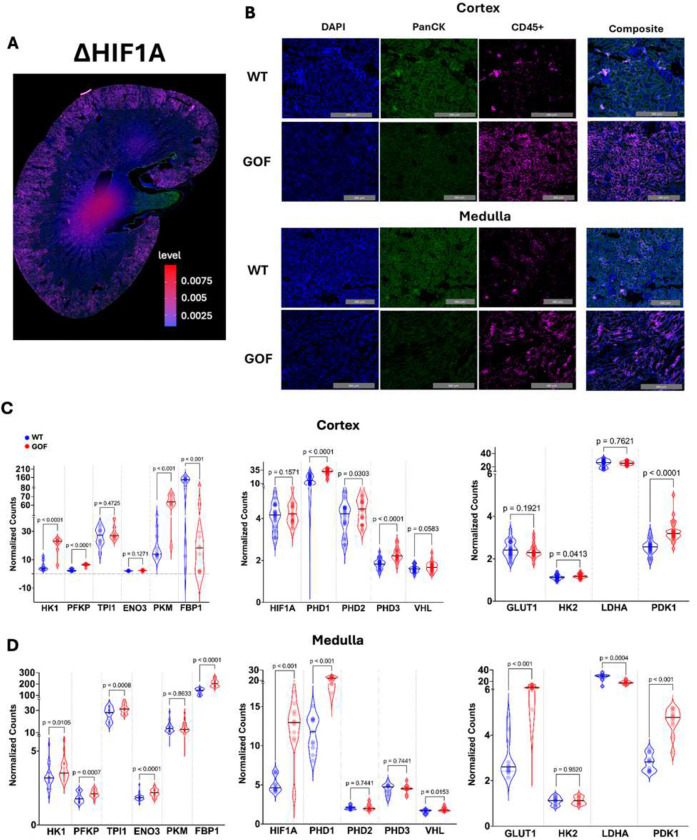
HSD-fed ENaC gain-of-function mice display significant HIF1A upregulation exclusively in renal medulla. (A) Differential expression of Hypoxia Inducible Factor 1-alpha (HIF1A) expressed as percent change in different pre-selected regions of interests (ROIs) probed for measurement of mRNA levels. (B) Representative images showing immunofluorescence staining using DAPI, PanCK, and CD45 antibody in the kidney of WT and HSD-fed ENaC GOF mice. (C) Expression levels of genes involved in glycolysis and HIF1A signaling in the ROIs found in the renal cortex of WT and GOF mice. (D) Expression levels of genes involved in glycolysis and HIF1A signaling in the ROIs found in the renal medulla of WT and GOF mice. Statistical comparisons between wild-type (WT) and gain-of-function (GOF) groups were performed using unpaired two-tailed Student’s t-tests. p values are displayed above each comparison; data are shown as mean ± SEM unless otherwise indicated.

Previously, our lab has demonstrated that excess interstitial sodium enters APCs via ENaC to stimulate oxidative stress, cytokine production, and T-cell activation in salt-sensitive hypertension^28,29,44–48^. We also found that a protein called SGK1 (serum/glucocorticoid kinase 1) modulates ENaC expression and assembly in APCs, promoting SSBP pathogenesis. Moreover, SGK1 ablation in CD11c^+^ cells reduced kidney inflammation, immune cell infiltration, and fibrosis^44^. Accordingly, we hypothesized that renal immune infiltrates display upregulation of glycolytic genes which is ENaC-dependent during SSBP progression. To study this, we utilized CRISPR/Cas9 to produce an ENaC gain-of-function mutant strain on the genetic background of salt-sensitive SV129 wild-type (WT) mice. These gene-edited mice possessed a single nucleotide variant (W521r) in the ENaC-alpha subunit which has been previously associated with human hypertensive disorders and BP dysregulation^49^. These mutant mice, alongside WT controls, were then fed HSD (4% NaCl) for three weeks and renal spatial transcriptomic analysis was subsequently performed. We identified twenty-four regions of interest (ROIs) in the kidneys of two WT mice and two ENaC mutant mice, classified them as either renal cortex or renal medulla ROIs, and examined differential expression within these ROIs following quality control and normalization. We found that ENaC gain-of-function mice exhibited significantly higher expression of the glycolytic genes HK1, PFKP, and PKM in the renal cortex while the renal medulla demonstrated significant increases of HK1, PFKP, TPI1, and ENO3 (Figure 6C-D). Interestingly, FBP1 (fructose bisphosphatase-1) was found to be significantly downregulated in the renal cortex; however, in the renal medulla, it was elevated (Figure 6C-D). This data suggests that sodium differentially affects glycolytic activity of immune cells in the cortex and medulla as FBP1 is a well-defined enzymatic regulator of glycolysis and gluconeogenesis.

### High sodium alters mitochondrial morphology

**Figure 7.**
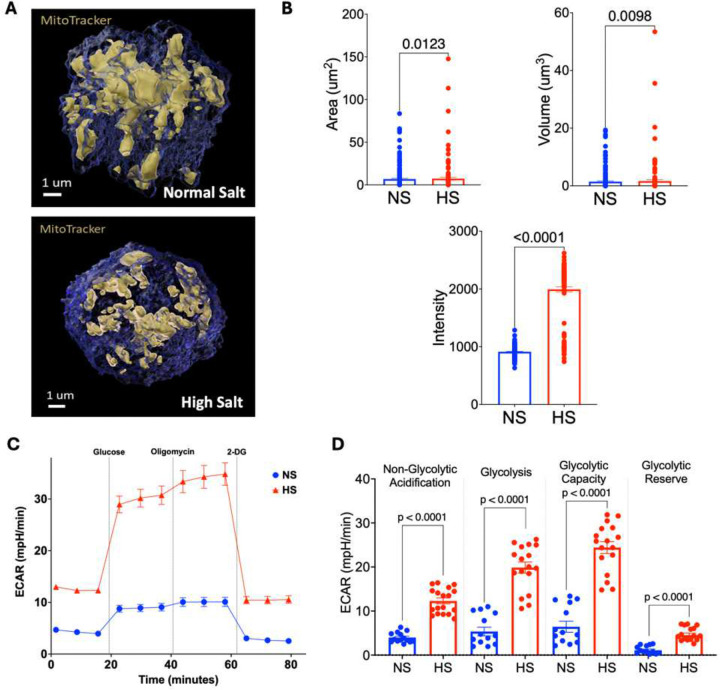
High sodium exposure significantly increases glycolytic activity in MHC class II+ APCs in vitro. (A) (B) (C) Glycolytic Stress Test results from Seahorse Extracellular Flux analysis following exposure to normal (150 mM) or excess (190 mM) sodium levels. (D) Non-glycolytic acidification, glycolysis, glycolytic capacity, and glycolytic reserves calculated from Glycolysis Stress Test results compared to their controls. Data are presented as mean ± SEM. Statistical significance between groups was determined using unpaired two-tailed Student’s t-tests. p values are shown for comparisons between normal salt (NS) and high salt (HS) conditions.

High sodium loading induces mitochondrial remodeling in MHC class II^+^ APCs, as demonstrated by the 3D reconstructions in Figure 5. Figures 7A and 7B illustrate that high sodium (HS) exposure induces marked structural remodeling of mitochondria in MHC class II^+^ antigen-presenting cells (APCs). Three-dimensional MitoTracker imaging (Figure 7A) shows that under normal salt (NS, 150 mM), mitochondria exhibit an extensive, interconnected network, fused mitochondrial state. In contrast, cells exposed to HS (190 mM) display fragmented and condensed mitochondria, suggesting enhanced mitochondrial fission or degradation. Quantitative metrics in Figure 7B reinforce these morphological observations: both mitochondrial area and volume are significantly reduced under HS conditions (p = 0.0123 and p = 0.0098, respectively), reflecting a loss of mitochondrial mass or integrity. Interestingly, despite this reduction, MitoTracker intensity is significantly elevated in HS-treated cells (p < 0.0001). This may reflect a hyperpolarized mitochondrial membrane or increased accumulation of dysfunctional mitochondria, both of which are consistent with cellular stress responses. These findings suggest that high sodium disrupts mitochondrial homeostasis, a key feature of metabolic reprogramming. By impairing mitochondrial structure and potentially altering function, sodium loading may drive APCs toward a glycolysis-dominant phenotype, contributing to the inflammatory and immunometabolic changes observed in salt-sensitive disease states.

### High sodium induces mitochondrial remodeling, glycolytic activation, and superoxide production.

**Figure 8.**
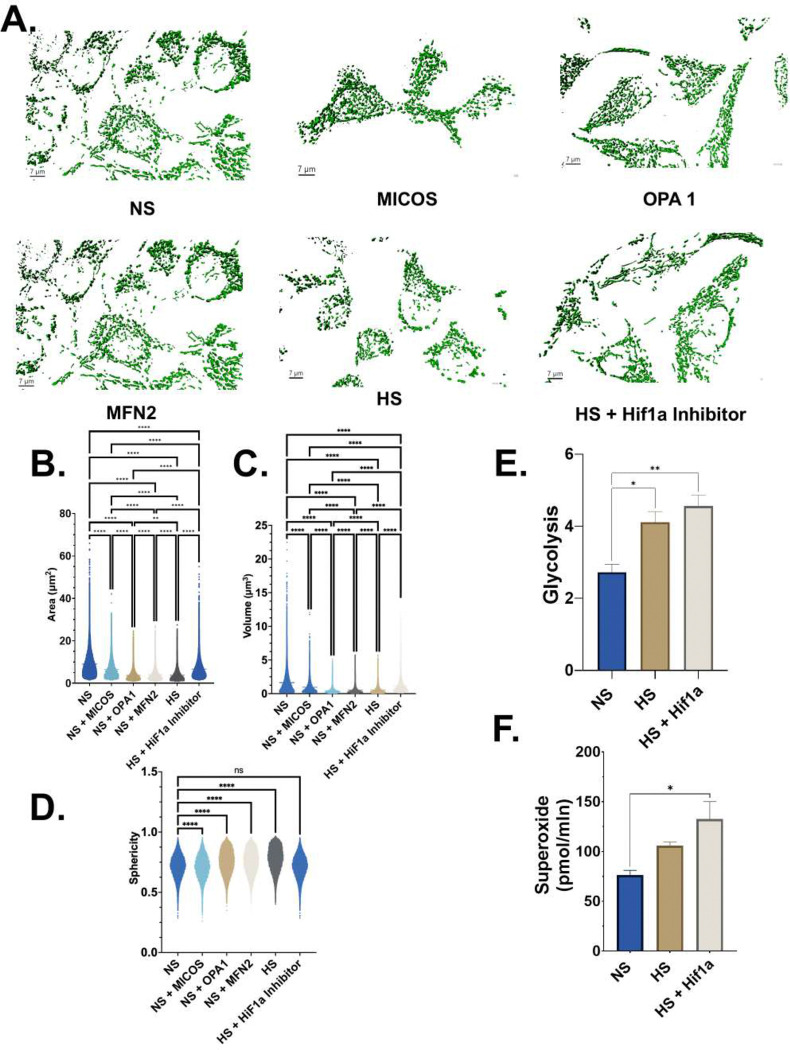
High sodium induces mitochondrial remodeling, glycolytic activation, and superoxide production. (A) Representative three-dimensional surface renderings of mitochondrial networks in cells under normal sodium (NS), following perturbation of mitochondrial structural regulators (MICOS, OPA1, or MFN2), high sodium (HS), or HS in the presence of a HIF-1α inhibitor. Mitochondria were imaged by spinning disk confocal and SoRa super-resolution microscopy and reconstructed in Imaris. Scale bars, 7 μm. (B) Quantification of mitochondrial surface area across experimental conditions. (C) Quantification of mitochondrial volume across experimental conditions. (D) Quantification of mitochondrial sphericity as a measure of mitochondrial morphology. (E) Glycolytic activity measured in cultured HeLa cells under NS, HS, and HS with HIF-1α inhibition. (F) Superoxide production measured by electron paramagnetic resonance (EPR) spectroscopy in cultured HeLa cells under NS, HS, and HS with HIF-1α inhibition. For panels B–D, violin plots surface areas reconstructed from three-dimensional images; horizontal lines indicate medians. Statistical comparisons were performed using appropriate multiple-comparison tests as indicated. For panels E–F, bars represent mean ± SEM. Significance is denoted as p < 0.05 (), p < 0.01 (**), and **p < 0.0001 (***); ns, not significant.

To determine the impact of sodium on mitochondrial structure, live-cell mitochondria were imaged using spinning-disk confocal microscopy and SoRa super-resolution microscopy, and the data were reconstructed into three-dimensional surfaces for quantitative morphometric analysis. Under normal sodium conditions, mitochondria formed elongated, interconnected networks with extensive branching (Figure. 8A). High sodium treatment markedly disrupted mitochondrial organization, producing shorter, fragmented structures with reduced network continuity (Figure. 8A). Perturbation of mitochondrial structural regulators (MICOS, OPA1, or MFN2) under NS conditions recapitulated the HS phenotype, resulting in fragmented mitochondria and loss of elongated networks (Figure. 8A). Inhibition of HIF-1α during HS partially restored mitochondrial elongation and network organization relative to HS alone (Figure. 8A). Quantitative analysis demonstrated a significant reduction in mitochondrial surface area following HS exposure compared with NS (Figure. 8B). Similar reductions were observed following MICOS, OPA1, or MFN2 perturbation under NS conditions (Figure. 8B). HIF-1α inhibition during HS significantly increased mitochondrial surface area relative to HS alone, although values remained below NS levels (Figure. 8B). Mitochondrial volume was significantly decreased under HS conditions compared with NS and was similarly reduced following MICOS, OPA1, or MFN2 perturbation under NS conditions (Figure. 8C). Treatment with a HIF-1α inhibitor during HS significantly increased mitochondrial volume relative to HS alone (Figure. 8C). Mitochondrial sphericity was significantly increased under HS conditions, consistent with mitochondrial fragmentation, and was similarly elevated following MICOS, OPA1, or MFN2 perturbation under NS conditions (Figure. 8D). In contrast to surface area and volume, HIF-1α inhibition during HS did not significantly reduce mitochondrial sphericity compared with HS alone (Figure. 8D). To assess whether these structural changes were accompanied by alterations in cellular metabolism, glycolytic activity and reactive oxygen species production were measured in cultured HeLa cells (Figure. 8E–F). HS exposure significantly increased glycolytic activity compared with NS, and glycolysis was further elevated following HIF-1α inhibition during HS (Figure. 8E). In parallel, electron paramagnetic resonance analysis revealed a significant increase in superoxide production under HS conditions compared with NS, with the highest levels observed in cells treated with HS in the presence of a HIF-1α inhibitor (Figure. 8F).

### High sodium alters the spatial distribution of mitochondria in a HIF-1α–dependent manner

**Figure 9.**
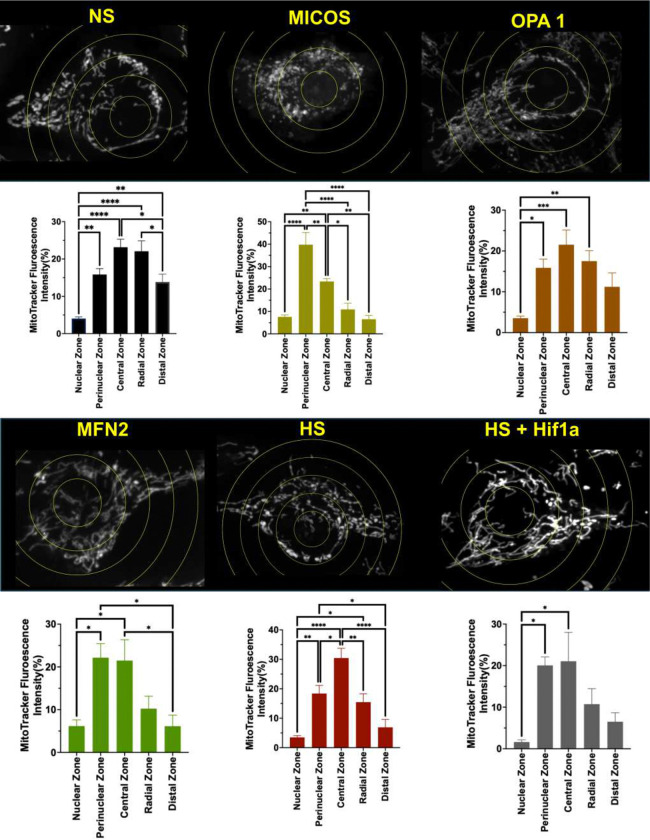
Impact of high sodium on intracellular mitochondrial distribution. Representative fluorescence images of mitochondria labeled with MitoTracker and analyzed using a concentric ring–based approach to quantify intracellular mitochondrial localization. Concentric regions were defined relative to the nucleus and segmented into nuclear, perinuclear, central, radial, and distal cytoplasmic zones (yellow rings). Top and bottom panels show representative images and corresponding quantitative analyses for cells under normal sodium (NS), perturbation of mitochondrial structural regulators (MICOS, OPA1, or MFN2), high sodium (HS), and HS in the presence of a HIF-1α inhibitor. Bar graphs depict the percentage of total MitoTracker fluorescence intensity within each concentric zone for the indicated condition. Data are presented as mean ± SEM. Statistical comparisons between zones and conditions were performed using appropriate multiple-comparison tests as indicated. Significance is denoted as p < 0.05 (), p < 0.01 (**), p < 0.001 (), and *p < 0.0001 (**).

To determine whether increased sodium exposure alters mitochondrial localization, cells from the previous experiment were reanalyzed using a concentric-ring-based approach in FIJI. MitoTracker fluorescence intensity was quantified as the sum of integrated densities across defined nuclear, perinuclear, central, radial, and distal cytoplasmic zones to assess mitochondrial localization relative to the nucleus. Under normal sodium conditions, mitochondria exhibited a broad spatial distribution, with fluorescence intensity enriched in central and radial cytoplasmic regions and low signal within the nuclear and perinuclear zones (Figure.9A). In contrast, disruption of MICOS resulted in a pronounced redistribution of mitochondria toward the perinuclear and central regions, with a significant reduction in radial and distal mitochondrial signal (Figure. 9B). Perturbation of OPA1 similarly shifted mitochondrial fluorescence toward the perinuclear and central zones, accompanied by decreased distal localization (Figure. 9C). MFN2 perturbation also altered mitochondrial spatial organization, increasing perinuclear and central enrichment while reducing mitochondrial signal in more distal regions (Figure. 9D). HS exposure induced a marked redistribution of mitochondria toward the perinuclear and central zones compared with NS, with a corresponding reduction in radial and distal mitochondrial fluorescence (Figure. 9E). This spatial pattern closely resembled that observed following perturbation of mitochondrial structural regulators, indicating that HS promotes perinuclear mitochondrial accumulation. Inhibition of HIF-1α during HS exposure partially reversed this redistribution. Compared with HS alone, HIF-1α inhibition reduced perinuclear and central mitochondrial enrichment and increased mitochondrial signal in radial and distal regions, although the spatial distribution did not fully return to NS levels (Figure. 9F).

### High-Salt Diet Impairs Locomotor Performance, Disrupts Mitochondrial Ultrastructure, and Induces End-Organ Remodeling in Drosophila melanogaster

**Figure 10.**
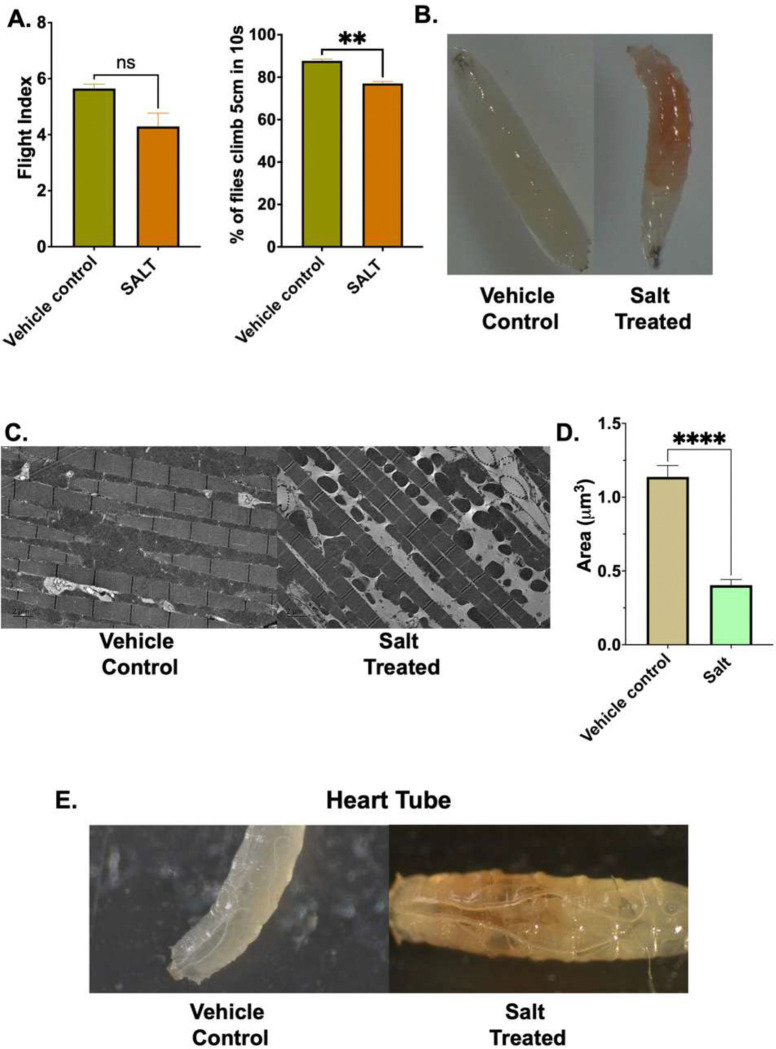
High-salt diet impairs locomotor performance, induces intestinal pigmentation, disrupts flight muscle ultrastructure, and alters cardiac morphology in Drosophila. (A) Quantification of flight performance using a vertical drop assay (left), expressed as flight index, and negative geotaxis climbing ability (right), expressed as the percentage of flies reaching 5 cm within 10 seconds, in vehicle control and salt-treated flies. (B) Representative gross morphological images of dissected larva preparations from vehicle control and salt-treated flies. (C) Representative transmission electron micrographs of indirect flight muscle from vehicle control and salt-treated flies (scale bar = 2 μm). (D) Quantification of mitochondrial cross-sectional area (μm^²^) in vehicle control and salt-treated flies. (E) Representative macroscopic images of heart tubes from vehicle control and salt-treated flies. Data are presented as mean ± SEM. Statistical comparisons were made using an unpaired two-tailed Student’s t-test. ns = not significant, ** p < 0.01, **** p < 0.0001.

To determine whether high-sodium-induced pathophysiology extends beyond mammalian systems, we exposed Drosophila melanogaster to a high-salt diet and assessed locomotor performance, tissue morphology, and mitochondrial integrity. Salt-treated flies exhibited a significant reduction in negative geotaxis climbing ability compared to vehicle controls, with a non-significant trend toward lowered flight index, indicating impaired locomotor performance following sodium excess (Figure 10A). Gross morphological analysis of whole-body larvae revealed increased pigmentation in salt-treated animals compared to vehicle controls. This darkening is consistent with stress-induced melanization and likely reflects oxidative stress and tissue damage. Dissection further showed marked pigmentation in salt-treated larvae, suggesting compromised barrier integrity. (Figure 10B). Given the mitochondrial remodeling observed in human immune cells under high sodium, we next examined flight muscle ultrastructure by transmission electron microscopy. Salt-treated flies exhibited pronounced mitochondrial dysmorphology characterized by loss of cristae integrity and organelle disorganization compared to the densely packed, well-ordered mitochondria observed in vehicle controls (Figure 10C). Quantification of mitochondrial cross-sectional area confirmed a significant reduction in salt-treated animals relative to controls (Figure 10D). Finally, macroscopic examination of dissected heart tubes revealed gross morphological and pigmentation changes in salt-treated flies compared to vehicle controls (Figure 10E), consistent with sodium-induced cardiac remodeling. Collectively, these findings demonstrate that the physiological consequences of sodium excess are conserved across species and manifest independently of an adaptive immune system or closed circulatory system, underscoring the cell-autonomous nature of sodium-induced mitochondrial and end-organ dysfunction.

## Discussion

This study identifies a coordinated, HIF1α-dependent axis of mitochondrial metabolic adaptation that is mechanistically distinct from a generalized stress response to excess dietary sodium. This response is defined by convergent evidence spanning reorganization of circulating TCA metabolites, transcriptional suppression of oxidative phosphorylation, chromatin-level HIF1α activation, in vivo renal validation, and structural remodeling of the mitochondrial network. Its coherence across modalities, tissues, and species establishes mechanistic depth and supports broader physiological relevance to salt-sensitive blood pressure regulation. At the population level, PheWAS and LabWAS analyses in the All of Us Research Program independently corroborated this framework, identifying renal and electrolyte phenotypes as the strongest hypertension-associated disease categories and linking hypertension to reduced serum potassium, chloride, and eGFR, consistent with the renal-electrolyte axis central to sodium-driven blood pressure dysregulation.

The metabolomic data establish that sodium-driven mitochondrial flux changes are systemic and hemodynamically coupled. Within-subject sodium loading and depletion produced non-overlapping plasma metabolome clusters by principal component analysis, and selective accumulation of succinate, fumarate, succinylcarnitine, and related intermediates under sodium loading was mirrored by suppression of upstream TCA enzyme transcripts, including IDH2, fumarate hydratase, and SDHD, in the transcriptomic dataset. This correspondence between metabolite accumulation and gene suppression of enzymes identifies a transcriptionally driven bottleneck at the succinyl-CoA-to-succinate node. Succinate-mediated prolyl hydroxylase inhibition provides a non-hypoxic mechanism for HIF1α stabilization, closing a feedforward loop in which sodium-induced HIF1α activation suppresses TCA flux, succinate accumulates, and prolyl hydroxylase inhibition further amplifies HIF1α activity. Elevated reactive oxygen species production from remodeled mitochondria likely contributes an additional stabilizing input to this loop. Quantitative correlations between changes in individual TCA metabolites and changes in systolic blood pressure, diastolic blood pressure, pulse pressure, and mean arterial pressure establish that this mitochondrial metabolic state is not a parallel observation but a measurable predictor of the vascular response to salt, directly supporting the cardiovascular relevance of the pathway.

The transcriptomic architecture of the sodium response reflects active metabolic coordination rather than a nonspecific shutdown of oxidative function. High-sodium conditions produced broad downregulation of oxidative phosphorylation genes spanning Complex I, III, and IV subunits alongside robust induction of canonical HIF1α glycolytic targets, including ENO3, FBP1, HK1, and PKM. The breadth of complex-spanning OXPHOS suppression distinguishes this from a localized enzymatic defect and supports a coordinated, transcription-factor-driven remodeling of respiratory capacity. The pyruvate dehydrogenase complex regulatory axis provided the most direct evidence of active coordination: concurrent downregulation of PDK2, PDK3, and PDK4 with upregulation of PDP1 and PDP2 reflects fine-tuned adjustment of carbon routing at the pyruvate node that would not be expected from a passive stress response. The bidirectional regulation of glycolytic gene expression by sodium availability, suppressed by in vivo sodium depletion and induced by excess sodium in vitro, confirmed that the transcriptional response is dynamically sodium-responsive and not a fixed metabolic difference between individuals.

Single-cell chromatin accessibility profiling extended the mechanistic framework to the epigenomic level and established a translational link to the blood pressure phenotype. HIF1α motif activity was enriched in specific immune cell clusters rather than distributed globally, consistent with cell-type-selective regulatory remodeling. Changes in HIF1α transcription factor activity correlated strongly with changes in systolic blood pressure and pulse pressure, with correlation coefficients exceeding 0.90 in both cases, demonstrating that chromatin-level HIF1α state in circulating immune cells is a quantitative predictor of hemodynamic salt sensitivity. Murine renal HIF1α gain-of-function provided in vivo causal confirmation that HIF1α activation is sufficient to drive the transcriptional response observed in human cells, with the strongest effects concentrated in the medulla, consistent with the spatial HIF1α expression gradient and the high energetic demands of this zone. Engagement of the full PHD and VHL negative-feedback circuit in the gain-of-function kidney indicated that HIF1α activation was not isolated to a single transcriptional node but propagated through the canonical regulatory axis.

The mitochondrial imaging and functional data establish the structural basis of the metabolic adaptation and identify HIF1α as causally upstream of network remodeling. High-sodium immune cells exhibited mitochondrial fragmentation with increased sphericity, along with a nearly twofold increase in MitoTracker fluorescence intensity, indicating increased mitochondrial mass or membrane potential rather than organellar degeneration. Overexpression of the fusion and cristae-organizing regulators MICOS, OPA1, and MFN2 individually phenocopied aspects of the high-sodium morphology under normal-salt conditions, confirming engagement of conserved structural pathways. Pharmacological inhibition of HIF1α in high-sodium cells reversed fragmentation. It produced a hyperfused, elongated network architecture, establishing that HIF1α activity is required to maintain the fragmented state and that its removal releases a tonically suppressed fusion state. This causal relationship between HIF1α and mitochondrial structure, together with the Seahorse data showing expansion of glycolytic capacity and reserve rather than flux alone, indicates that immune cells under high-sodium conditions have made a structural and enzymatic commitment to glycolytic metabolism. The persistence of elevated glycolysis and superoxide following HIF1α inhibition, despite morphological normalization, demonstrates that structural and metabolic arms of this adaptation are partially dissociable, suggesting that HIF1α-independent effectors contribute to sustaining the metabolic phenotype and represent a priority question for future mechanistic investigation.

Collectively, these findings position sodium as a metabolic stressor that engages a self-amplifying, HIF1α-centered regulatory axis simultaneously remodeling mitochondrial structure, transcriptional state, and metabolic flux in immune cells.^7,50^ This extends prior observations that sodium suppresses mitochondrial respiration in mononuclear phagocytes and that osmotic stress activates HIF1α independent of hypoxia by identifying the full regulatory architecture through which these effects are coordinated and by demonstrating their hemodynamic relevance.^51,52^ The detection of this response in circulating immune cells, its quantitative coupling to blood pressure responses, and its recapitulation in mouse kidney parenchyma together argue that HIF1α-dependent mitochondrial remodeling represents both a candidate mechanistic driver and a measurable biosignature of salt-sensitive hypertension. Concordantly, Drosophila melanogaster subjected to a high-salt diet recapitulated sodium-induced end-organ dysfunction across the locomotor, gastrointestinal, musculoskeletal, and cardiac systems, establishing the phylogenetic conservation of this response independent of adaptive immunity and providing cross-species validation of the sodium-mitochondrial axis described here.

Limitations of this study include modest sample sizes that constrain statistical power, the absence of normotensive comparators, lack of sex-stratified analyses, and the incomplete mechanistic resolution of the HIF1α-independent component of the metabolic phenotype. Future studies employing expanded cohorts, longitudinal sampling, and targeted genetic perturbation will be essential for defining the causal architecture, generalizability, and therapeutic tractability of sodium-driven immunometabolic remodeling in the context of blood pressure regulation.

## Supplementary Material

Supplement 1

## Figures and Tables

**Table 1.** Demographics and clinical characteristics of patients for Salt-Sensitivity Phenotyping Study. N = 30. SBP, Systolic Blood Pressure, DBP, Diastolic Blood Pressure, HTN, Hypertension, BMI, Body Mass Index, Data for continuous measures are presented as mean ± SD.

Age, years	53.1 ± 7.4
Male sex, n(%)	14(46.7)
Female sex, n(%)	16(53.3)
African American, n(%)	10(33.3)
Asian, n(%)	1(3.3)
White, n(%)	19(63.3)
Baseline SBP, mmHg	140.2 ± 14.6
Baseline DBP, mmHg	87.2 ± 9.4
HTN, n(%)	30(100)
BMI, kg/m^2^	32.1 ± 8.0

**Table 2.** Demographics and clinical characteristics of patients for Bulk Transcriptomic Analysis. N = 11. Systolic Blood Pressure, DBP, Diastolic Blood Pressure, HTN, Hypertension, BMI, Body Mass Index Data for continuous measures are presented as mean ± SD.

Age, years	34.7 ± 11.8
Female sex, n(%)	11(100)
African American, n(%)	1(9)
White, n(%)	10(91)
SBP, mmHg	110.9 ± 16.7
DBP, mmHg	69.0 ± 6.8
HTN, n(%)	11(100)
BMI, kg/m^2^	28.6 ± 17.5

## Data Availability

All data reported in this article will be made available by the corresponding author upon request. Experimental materials, methods, and data supporting the study findings are available in the article. Bulk RNA sequencing data for this study have been deposited on Figshare and are accessible to the public at https://figshare.com/s/d810937dc537eeb361a5. The data that support the findings of this study are available from the corresponding author upon reasonable request. To protect participant privacy, All of Us data used in this study are available only to registered researchers through the All of Us Researcher Workbench, which can be accessed via https://workbench.researchallofus.org/login, subject to institutional agreements (e.g., a Data Use and Registration Agreement), required training, and data use policy compliance. Analysis code can be shared with authorized All of Us Researcher Workbench users upon reasonable request
