# Supplementary material for: A sodium-HIF1α axis coordinates immune metabolic reprogramming and mitochondrial remodeling in salt-sensitive hypertension
Source: Res Sq. 2026 Apr 24:rs.3.rs-9504540. Preprint. [Version 1] doi: 10.21203/rs.3.rs-9504540/v1 (PMC13131874; doi:10.21203/rs.3.rs-9504540/v1)
Supplement: Supplement 1 [file NIHPPRS9504540V1-supplement-1.pdf]

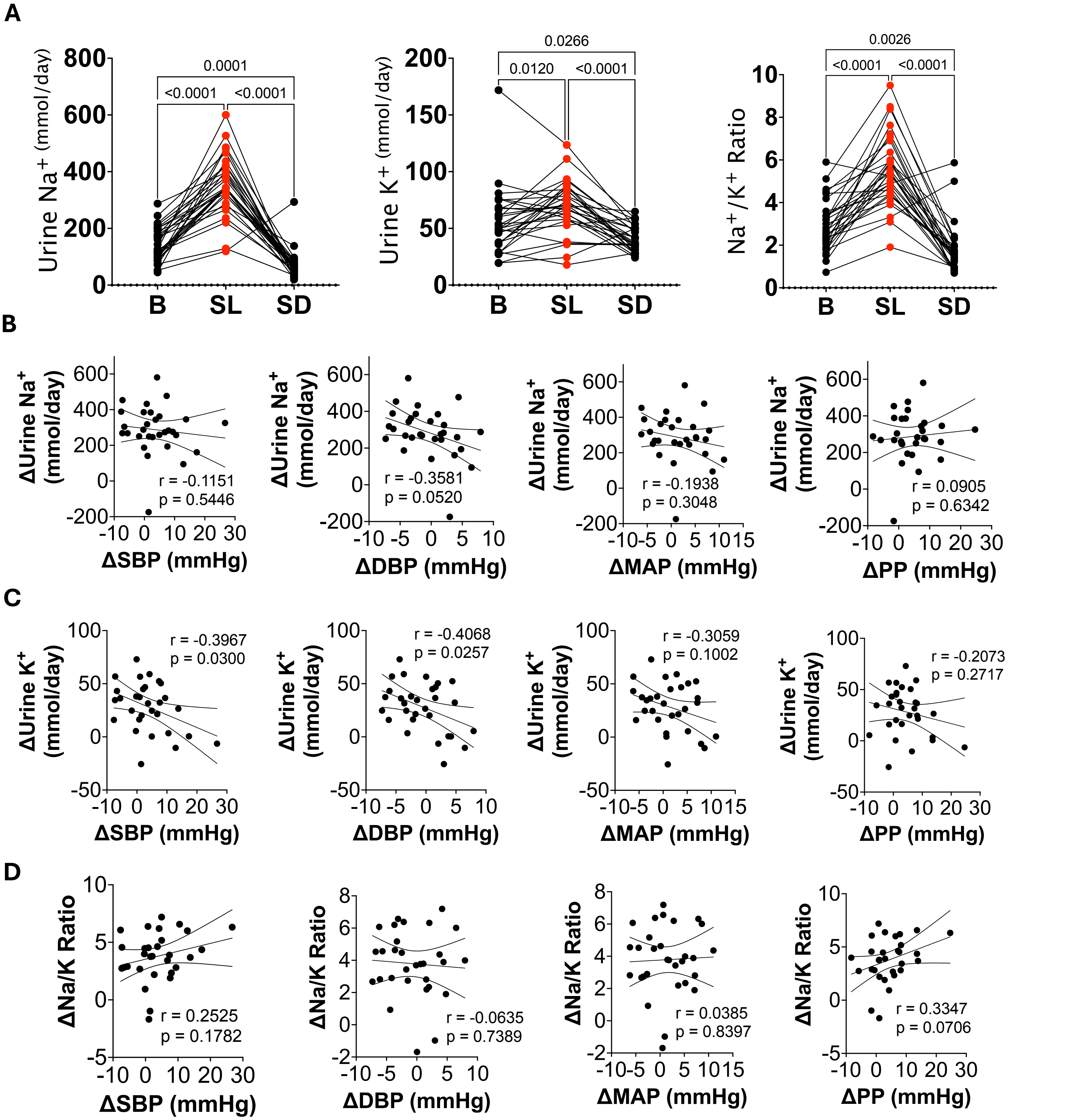

Supplementary Figure 1: Urinary sodium and potassium excretion across dietary salt conditions and their association with blood pressure responses. (A) Twenty-four–hour urinary sodium (Na^+), potassium (K^+), and Na^+/K^+ ratio were measured at baseline, during salt loading (SL), and during salt depletion (SD). Each line represents an individual participant. Group comparisons were analyzed using one-way repeated-measures ANOVA followed by Tukey's post hoc multiple-comparison correction; adjusted p

values are shown. (B–D) Associations between changes in urinary electrolyte excretion and changes in blood pressure across the dietary intervention. Correlations between Δurine Na^+ (B), Δurine K^+ (C), and $\Delta Na^+/K^+$ ratio (D) with changes in systolic blood pressure (ΔSBP), diastolic blood pressure (ΔDBP), mean arterial pressure (ΔMAP), and pulse pressure (ΔPP) were assessed using linear regression models, with Pearson correlation coefficients (r) and corresponding p values shown on each plot. Δ values represent the change between salt-loading and salt-depletion conditions.

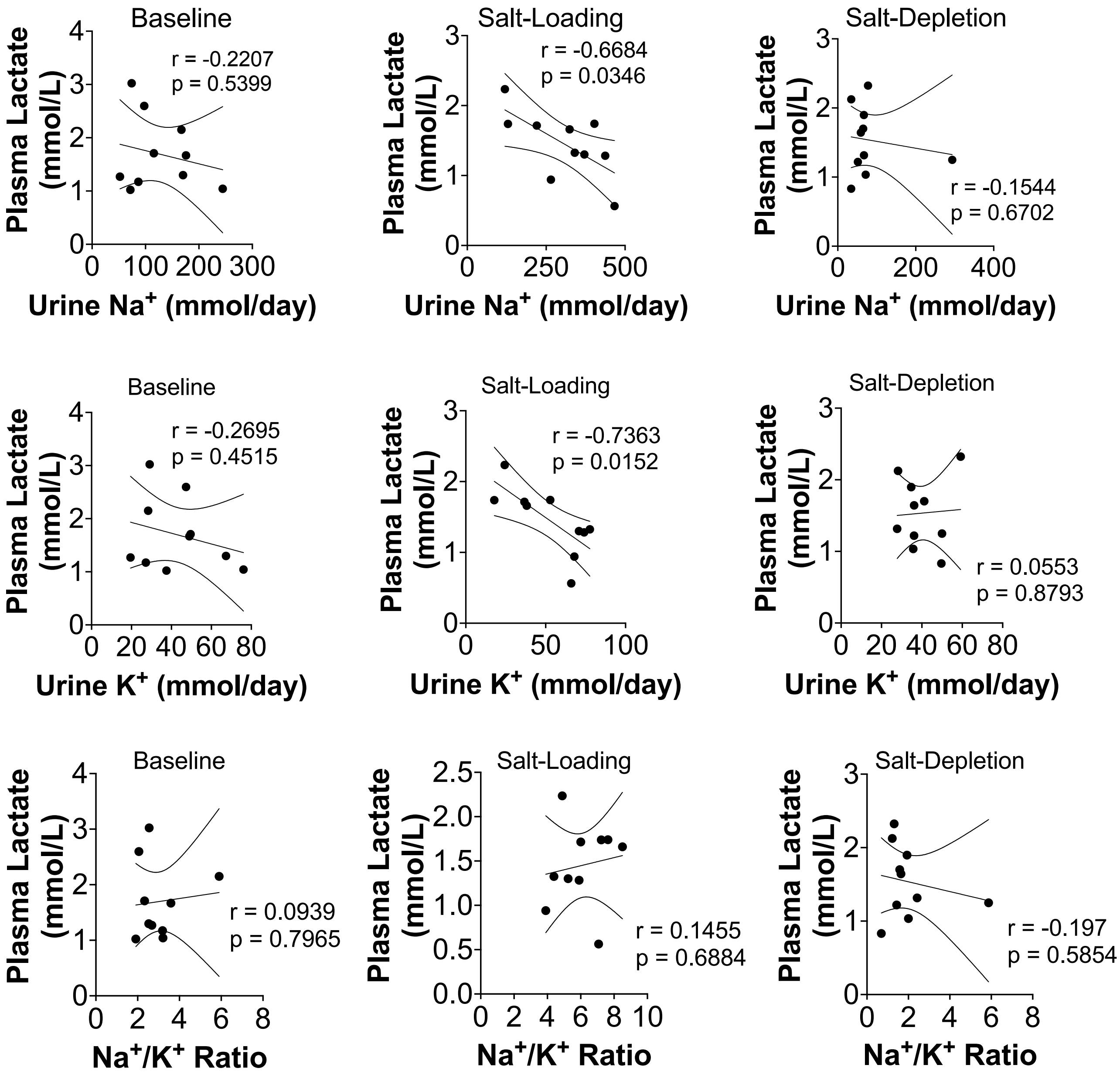

Supplementary Figure 2: Associations between urinary electrolyte excretion and plasma lactate across dietary salt conditions. Scatterplots show relationships between 24-hour urinary sodium (Na^+), potassium (K^+), and the Na^+/K^+ ratio, and plasma lactate concentrations at baseline, during salt loading, and during salt depletion. Associations were evaluated using linear regression models, with Pearson correlation coefficients and corresponding p values displayed on each plot. Lines represent the fitted regression with 95% confidence intervals.

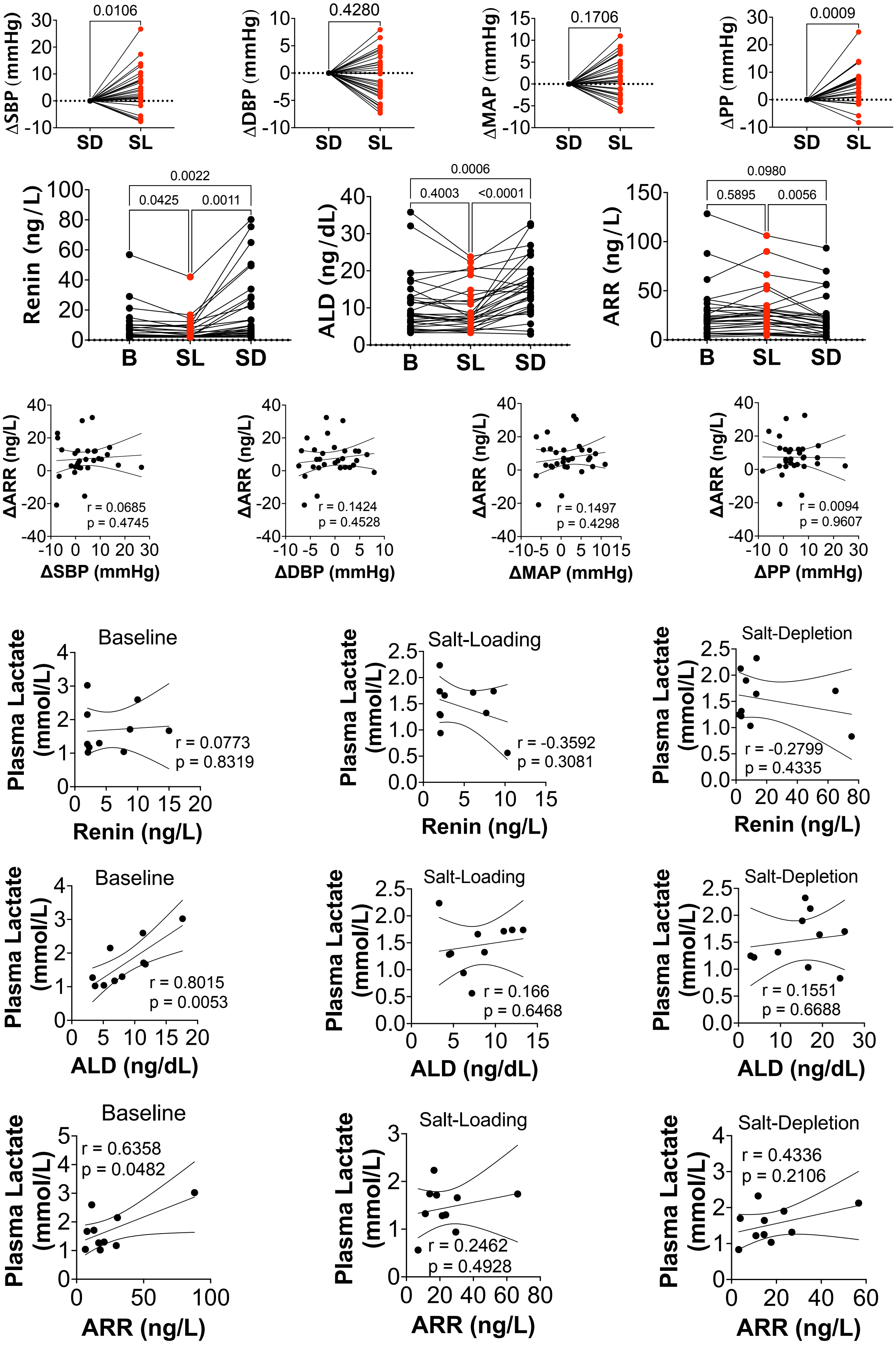

ΔSBP (mmHg)
0.0106
SD
SL
ΔDBP (mmHg)
0.4280
ΔMAP (mmHg)
0.1706
ΔPP (mmHg)
0.0009
Renin (ng/L)
0.0022
0.0425
0.0011
B
SL
SD
ALD (ng/dL)
0.0006
0.4003
<0.0001
ARR (ng/L)
0.0980
0.5895
0.0056
ΔARR (ng/L)
r = 0.0685
p = 0.4745
ΔSBP (mmHg)
r = 0.1424
p = 0.4528
ΔDBP (mmHg)
r = 0.1497
p = 0.4298
ΔMAP (mmHg)
r = 0.0094
p = 0.9607
ΔPP (mmHg)
Plasma Lactate (mmol/L)
Baseline
r = 0.0773
p = 0.8319
Renin (ng/L)
Salt-Loading
r = -0.3592
p = 0.3081
Salt-Depletion
r = -0.2799
p = 0.4335
Baseline
r = 0.8015
p = 0.0053
ALD (ng/dL)
Salt-Loading
r = 0.166
p = 0.6468
Salt-Depletion
r = 0.1551
p = 0.6688
Baseline
r = 0.6358
p = 0.0482
ARR (ng/L)
Salt-Loading
r = 0.2462
p = 0.4928
Salt-Depletion
r = 0.4336
p = 0.2106

Supplementary Figure 3: Blood pressure responses, renin–angiotensin–aldosterone system (RAAS) activity, and associations with plasma lactate across dietary salt conditions. Top panels show changes in systolic blood pressure (ΔSBP), diastolic blood pressure (ΔDBP), mean arterial pressure (ΔMAP), and pulse pressure (ΔPP) between salt depletion (SD) and salt loading (SL). Middle panels depict plasma renin, aldosterone (ALD), and aldosterone-to-renin ratio (ARR) measured at baseline (B), during salt loading (SL), and during salt depletion (SD). Each line represents an individual participant. Group comparisons were analyzed using one-way repeated-measures ANOVA followed by Tukey's post hoc multiple-comparison correction; adjusted p values are shown. Lower panels display associations between plasma lactate and renin, aldosterone, and ARR at baseline, during salt loading, and during salt depletion. Relationships were assessed using linear regression models with Pearson correlation coefficients (r) and corresponding p values indicated on each plot. Lines represent fitted regressions with 95% confidence intervals.

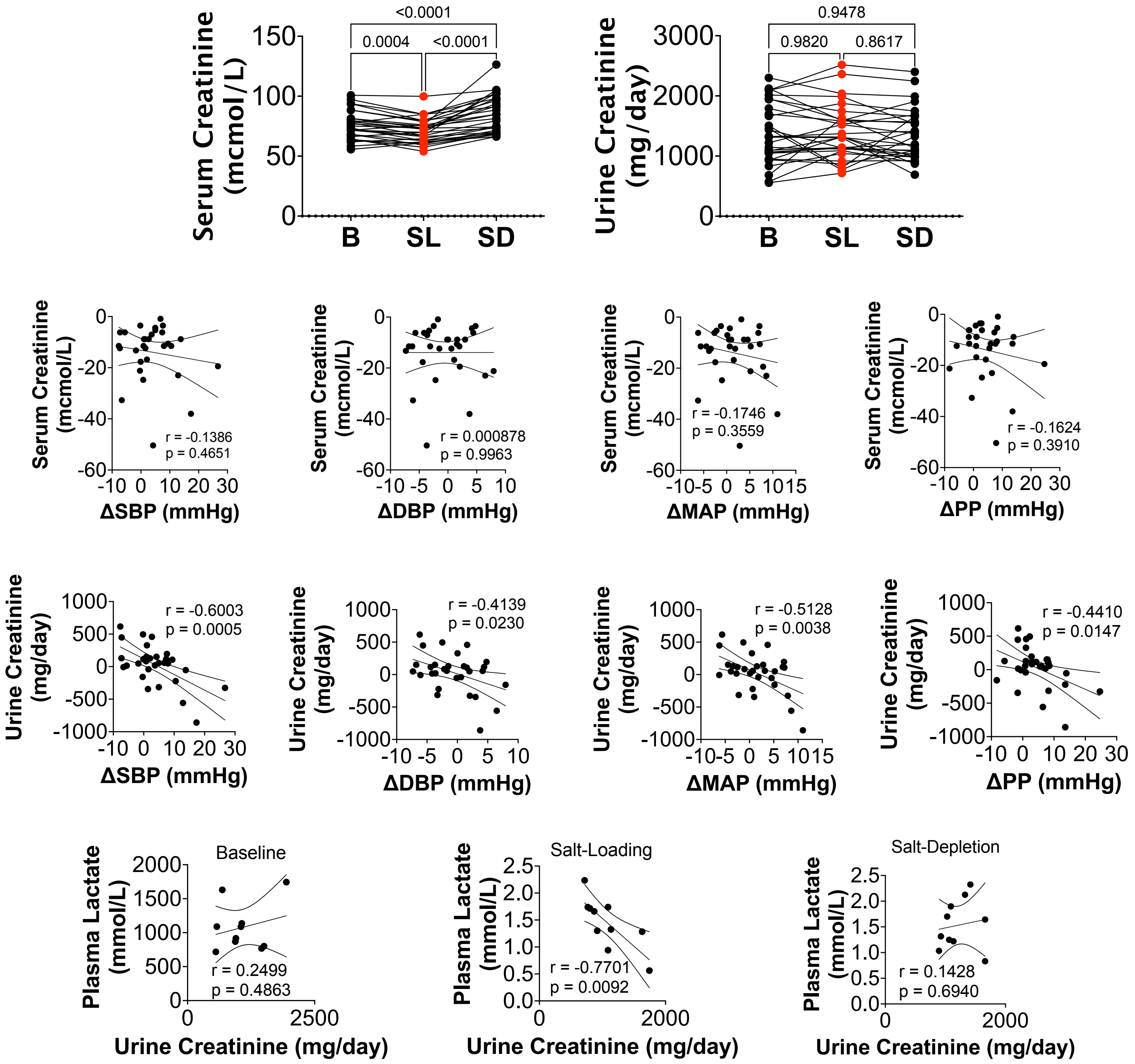

Supplementary Figure 4: Creatinine dynamics across dietary salt conditions and associations with blood pressure and plasma lactate. Top panels show serum creatinine and 24-hour urinary creatinine measured at baseline (B), during salt loading (SL), and during salt depletion (SD) (n = 30). Each line represents an individual participant. Group comparisons were analyzed using one-way repeated-measures ANOVA followed by Tukey's post hoc multiple-comparison correction; adjusted p values are shown. Middle panels display associations between changes in serum creatinine or urinary creatinine and changes in systolic blood pressure (ΔSBP), diastolic blood pressure (ΔDBP), mean arterial pressure (ΔMAP), and pulse pressure (ΔPP). Lower panels show associations between plasma lactate and urinary creatinine at baseline, during salt loading, and during salt depletion. Relationships were assessed using linear regression models with Pearson correlation coefficients (r) and corresponding p values indicated on each plot. Lines represent fitted regressions with 95% confidence intervals.

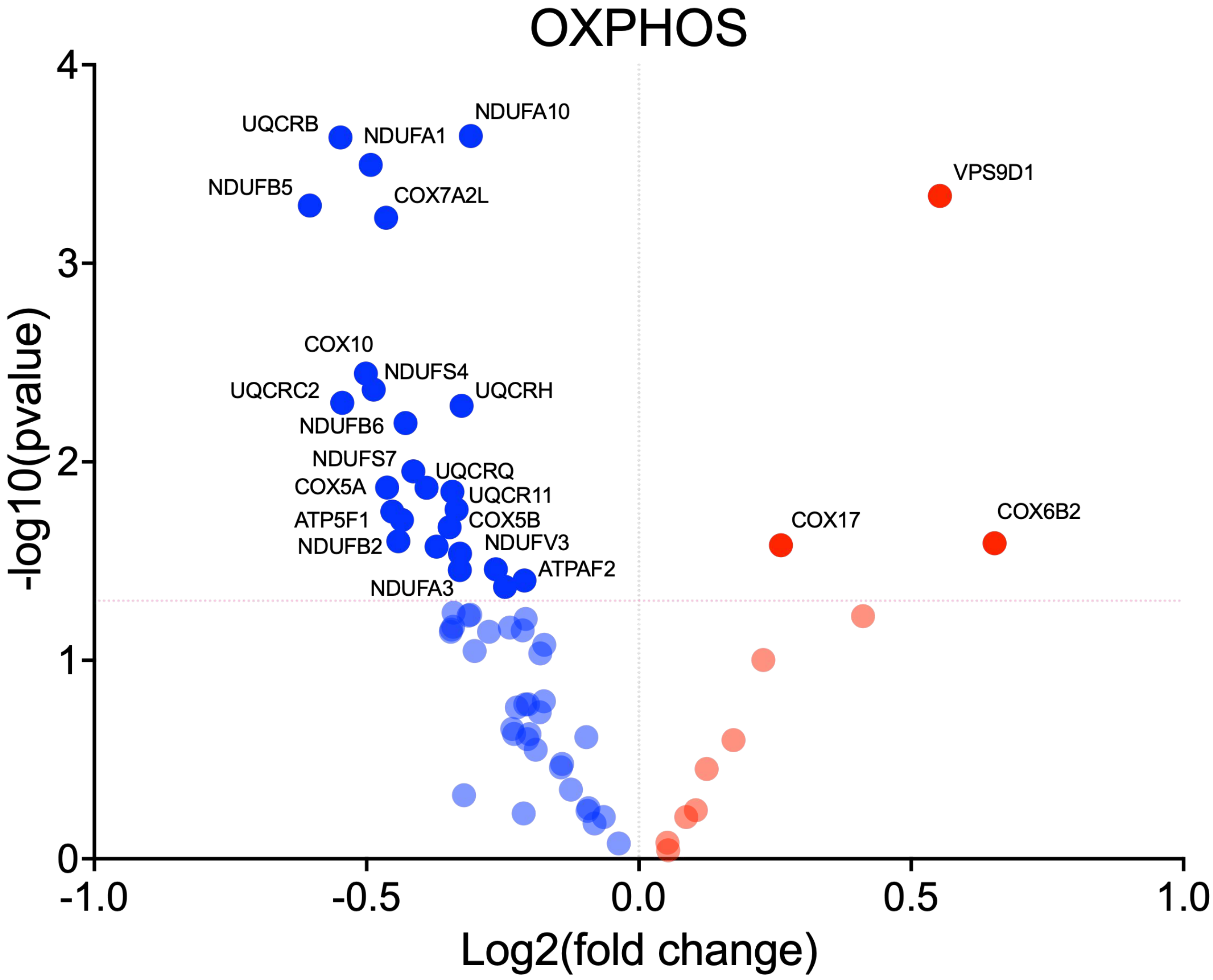

Supplementary Figure 5. Differential expression of OXPHOS-related genes under high-sodium conditions. Volcano plot depicting fold change (normal salt vs high salt) for OXPHOS-related transcripts. Genes enriched under normal salt are shown in blue and those enriched under high salt in red. The dashed line indicates the significance threshold (p = 0.05). Selected genes are labeled.

Supplementary Figure 6. Full clinical PheWAS of hypertension versus non-hypertensive controls in the *All of Us* cohort. Full clinical phenome-wide association study (PheWAS) of hypertension case status compared with non-hypertensive controls in the *All of Us* Research Program cohort. Each point represents a phecode tested in logistic regression, with hypertension case status modeled as the independent variable of interest and phecode case status as the dependent variable. ICD-9-CM and ICD-10-CM diagnosis codes were mapped to phecodes using phecode version 1.2. Models were adjusted for age at last EHR event, sex at birth, and the first five genetic principal components. Analyses were restricted to phecodes meeting prespecified minimum case thresholds. Phecodes are grouped by disease category on the *x*-axis, and the *y*-axis shows -$\log_{10}$(*p*-value). Upward triangles indicate phecodes associated with increased odds in hypertension cases, whereas downward triangles indicate phecodes associated with decreased odds in hypertension cases. The horizontal lines denote the nominal-significance threshold and the Bonferroni-correction threshold. Selected nominally significant and Bonferroni-significant phecodes are annotated.

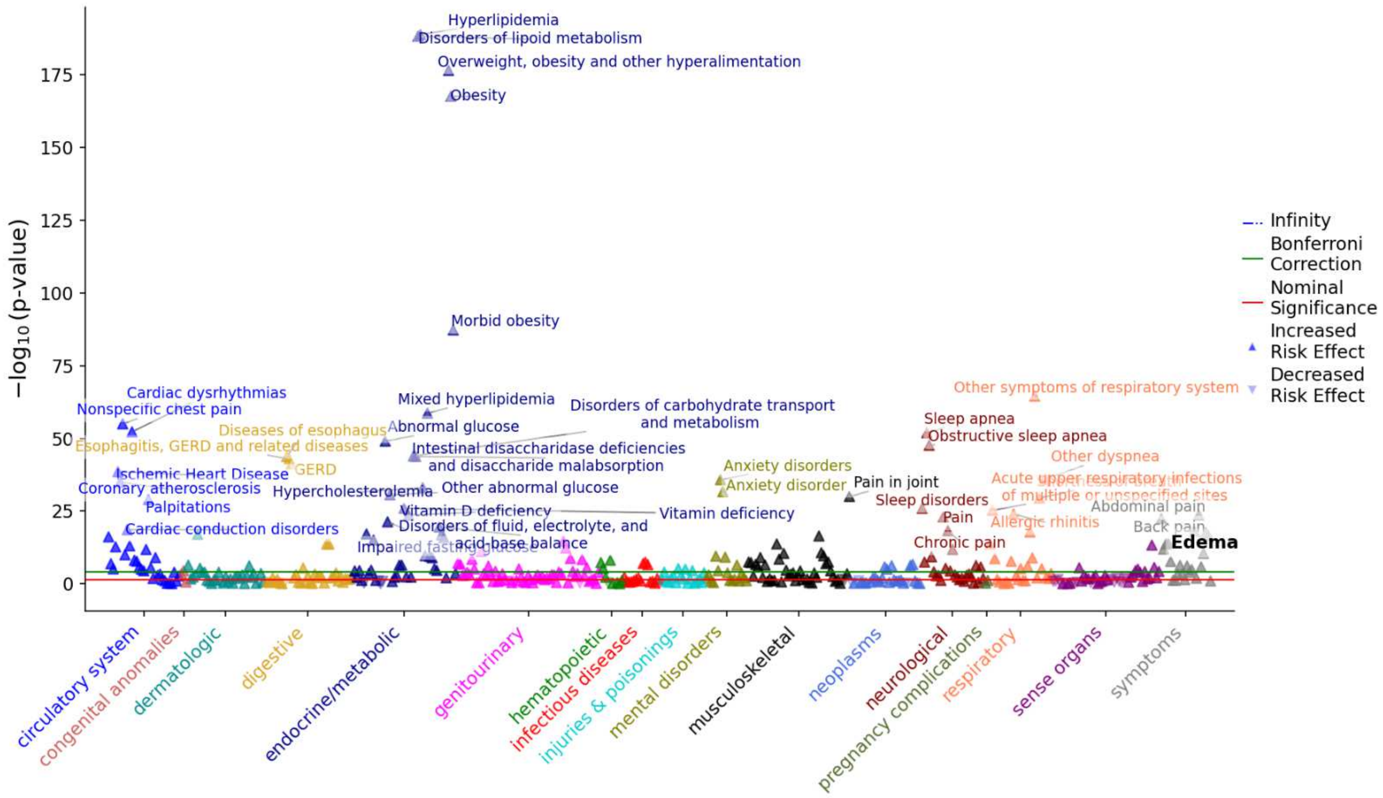

Supplementary Figure 7. Full clinical LabWAS of hypertension versus non-hypertensive controls in the *All of Us* cohort. Each point represents a harmonized laboratory phenotype tested in covariate-adjusted linear regression, with hypertension case status modeled as the independent variable of interest. Repeated measurements were summarized at the participant level; laboratory values were rank-based inverse normal transformed; and models were adjusted for age at the last EHR event, sex at birth, and the first five genetic principal components. Laboratory phenotypes are grouped by laboratory domain on the *x*-axis, and the *y*-axis shows -$\log_{10}$(*p*-value). Upward triangles denote laboratory phenotypes higher in hypertension cases, and downward triangles denote laboratory phenotypes lower in hypertension cases. Horizontal lines indicate the nominal-significance and Bonferroni-correction thresholds. Selected nominally significant and Bonferroni-significant laboratory phenotypes are annotated.

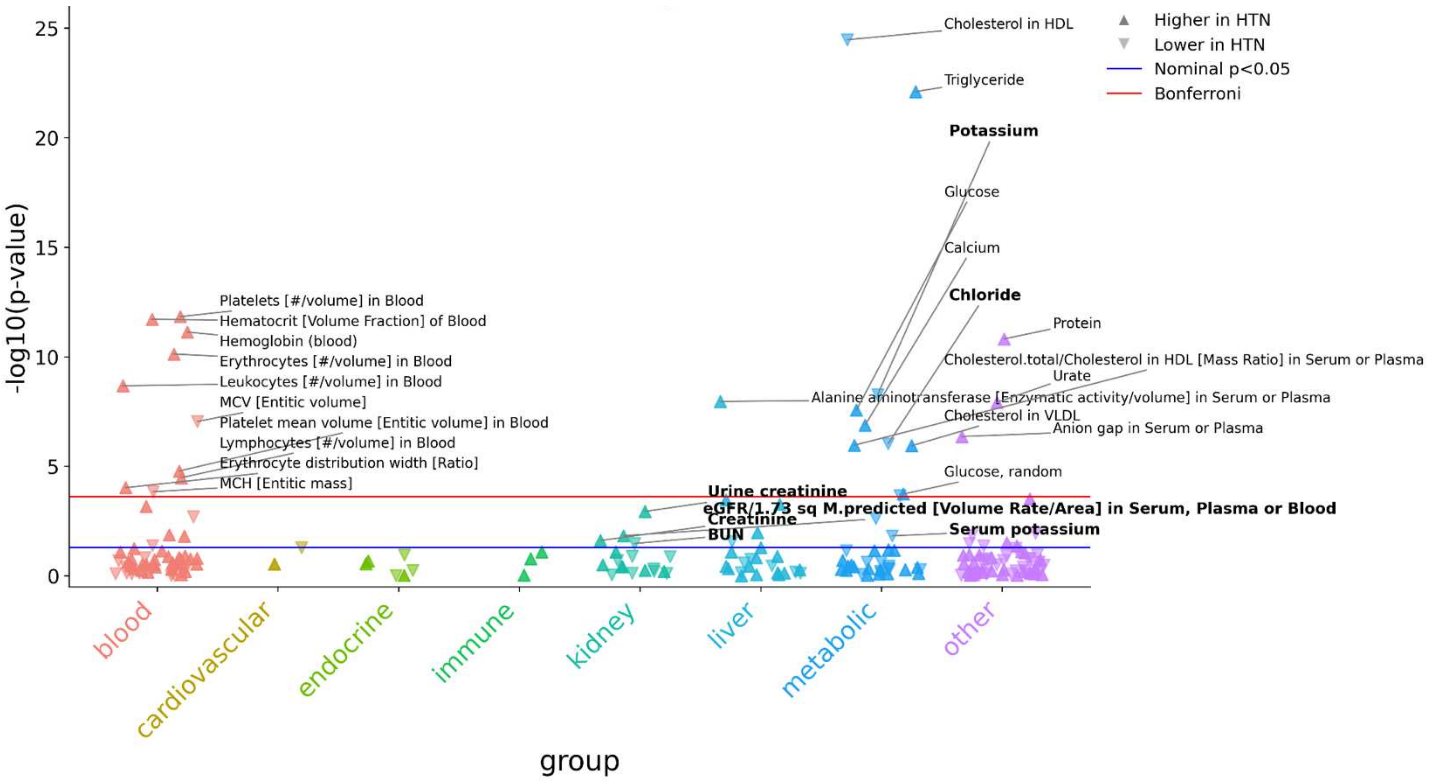

Characteristic	Controls (No HTN) (n = 45,710)	Hypertension (n = 4,187)
Age, years	41.3 ± 14.0	47.9 ± 12.0
**Sex, n (%)**		
Male	19,759 (43.2)	1,996 (47.7)
Female	25,157 (55.0)	2,160 (51.6)
**Race/ethnicity, n (%)**		
African American	10,473 (22.9)	1,175 (28.1)
Asian	2,882 (6.3)	179 (4.3)
White	18,326 (40.1)	1,831 (43.7)
SBP, mmHg	122.8 ± 16.6	137.8 ± 19.5
DBP, mmHg	77.3 ± 11.5	84.8 ± 13.1
HTN, n (%)	0 (0.0)	4,187 (100.0)
BMI, kg/m²	28.9 ± 40.6	33.5 ± 88.9

Supplementary Table 1. Demographics and clinical characteristics of patients for PheWAS and LabWAS in the All of Us Research Program. Systolic Blood Pressure, DBP, Diastolic Blood Pressure, HTN, Hypertension, BMI, Body Mass Index Data for continuous measures are presented as mean ± SD.

Phecode	Phenotype	Cases	Controls	OR (95% CI)	*p*-value	Direction
276	Disorders of fluid, electrolyte, and acid-base balance	161	26,898	4.91	3.01e-20	Higher in HTN
782.3	Edema	160	26,918	4.53	9.77e-19	Higher in HTN
276.1	Electrolyte imbalance	130	27,042	5.08	1.52e-17	Higher in HTN
586	Other disorders of the kidney and ureters	92	27083	3.19	8.90e-08	Higher in HTN
594.1	Calculus of kidney	142	27046	2.35	8.51e-06	Higher in HTN

Supplementary Table 2. Selected clinically relevant PheWAS associations for hypertension versus non-hypertensive controls in the *All of Us* Research Program. Each row represents a phecode-defined phenotype identified in the clinical hypertension case-control PheWAS in the *All of Us* Research Program. Hypertension case status was modeled as the independent variable of interest and phecode case status as the outcome in covariate-adjusted logistic regression, with adjustment for age at last EHR event, sex at birth, and the first five genetic principal components. Cases and controls indicate the numbers of participants in the analytic cohort with and without the corresponding phecode. Odds ratios (ORs) are shown for the association with hypertension case status, p-values and the direction of association. This table highlights selected phecodes related to salt-, electrolyte-, edema-, and kidney-related conditions prioritized for interpretation.

Supplementary Table 3. Bonferroni- and nominally significant laboratory phenotypes associated with hypertension versus non-hypertensive controls in the *All of Us* Research Program clinical LabWAS. Each row represents a laboratory phenotype identified in the clinical hypertension case-control LabWAS in the *All of Us* Research Program. Hypertension case status was modeled as the independent variable of interest in covariate-adjusted linear regression, with adjustment for age at last EHR event, sex at birth, and the first five genetic principal components. Laboratory phenotypes were harmonized across related measurement concepts, repeated measurements were summarized at the participant level, and values were rank-based inverse normal transformed before testing. Cases and controls indicate the numbers of hypertension cases and non-hypertensive controls with the corresponding laboratory phenotype available for analysis. Reported values include nominal *p* values and the direction of association, indicating whether the laboratory phenotype was higher or lower in hypertension. This table includes laboratory phenotypes Bonferroni- or nominal-significance thresholds in the full clinical LabWAS.

Group	Phenotype	Cases	Contro ls	*p*-value	Direction
other	Systolic blood pressure	3850	23133	0.00e+00	Higher in HTN
other	Computed systolic blood pressure, mean of 2nd and 3rd measures	3684	22502	7.35e-288	Higher in HTN
liver	Diastolic blood pressure	3861	23143	1.32e-263	Higher in HTN
liver	Computed diastolic blood pressure, mean of 2nd and 3rd measures	3697	22513	6.75e-228	Higher in HTN
other	Systolic blood pressure by palpation	168	444	3.73e-27	Higher in HTN
metabolic	Cholesterol in HDL	1254	4351	3.46e-25	Lower in HTN
metabolic	Triglyceride	1253	4370	8.07e-23	Higher in HTN
liver	Intravascular diastolic|Pressure|Moment in time|Without specimen	57	301	1.51e-16	Higher in HTN
blood	Platelets [#/volume] in Blood	1255	4966	1.48e-12	Higher in HTN
blood	Hematocrit [Volume Fraction] of Blood	2070	7369	1.97e-12	Higher in HTN
blood	Hemoglobin (blood)	1949	6755	7.51e-12	Higher in HTN

Group	Phenotype	Cases	Controls	*p*-value	Direction
other	Protein	1769	5726	1.55e-11	Higher in HTN
blood	Erythrocytes [#/volume] in Blood	1205	4752	7.55e-11	Higher in HTN
liver	Diastolic blood pressure--sitting	53	776	1.06e-09	Higher in HTN
blood	Leukocytes [#/volume] in Blood	1168	4378	2.13e-09	Higher in HTN
metabolic	Potassium	1773	6337	5.34e-09	Lower in HTN
other	Systolic blood pressure--sitting	52	775	5.80e-09	Higher in HTN
liver	Alanine aminotransferase [Enzymatic activity/volume] in Serum or Plasma	1051	4007	1.11e-08	Higher in HTN
other	Urate	98	242	1.22e-08	Higher in HTN
metabolic	Glucose	1766	6514	2.78e-08	Higher in HTN
blood	MCV [Entitic volume]	2064	7299	9.06e-08	Lower in HTN
metabolic	Calcium	1748	6351	1.34e-07	Higher in HTN
other	Anion gap in Serum or Plasma	777	2554	4.41e-07	Higher in HTN
metabolic	Chloride	1885	6526	9.43e-07	Lower in HTN
metabolic	Cholesterol.total/Cholesterol in HDL [Mass Ratio] in Serum or Plasma	267	1043	1.10e-06	Higher in HTN
metabolic	Cholesterol in VLDL	394	1273	1.16e-06	Higher in HTN
other	Sitting systolic blood pressure	24	191	3.80e-06	Higher in HTN
liver	Sitting diastolic blood pressure	24	191	7.52e-06	Higher in HTN
blood	Platelet mean volume [Entitic volume] in Blood	1439	4950	1.64e-05	Higher in HTN
blood	Lymphocytes [#/volume] in Blood	640	2703	3.33e-05	Higher in HTN
blood	Erythrocyte distribution width [Ratio]	1756	6588	9.46e-05	Higher in HTN
blood	MCH [Entitic mass]	1518	6022	1.45e-04	Lower in HTN
metabolic	Glucose, random	151	214	1.86e-04	Higher in HTN
metabolic	Serum HDL cholesterol	292	402	2.22e-04	Lower in HTN
other	Globulin [Mass/volume] in Serum	388	1369	3.06e-04	Higher in HTN
liver	Alanine aminotransferase [Enzymatic activity/volume] in Serum or Plasma by No addition of P-5'-P	156	321	3.42e-04	Higher in HTN
other	Mean blood pressure	79	177	4.84e-04	Higher in HTN
liver	Alkaline phosphatase [Enzymatic activity/volume] in Serum or Plasma	1272	4562	5.37e-04	Higher in HTN
blood	Neutrophils [#/volume] in Blood	593	2400	6.65e-04	Higher in HTN
kidney	Creatinine (urine)	213	410	1.15e-03	Higher in HTN
blood	Platelet mean volume [Entitic volume] in Blood by Rees-Ecker	196	1028	2.00e-03	Lower in HTN
metabolic	25-hydroxyvitamin D3	296	1137	2.40e-03	Lower in HTN
liver	Albumin by Bromocresol green (BCG) dye binding method	304	832	1.05e-02	Higher in HTN
other	Iron	174	667	1.13e-02	Lower in HTN
blood	Monocytes [#/volume] in Blood	629	2676	1.34e-02	Higher in HTN
other	Iron saturation [Mass Fraction] in Serum or Plasma	134	550	1.46e-02	Lower in HTN
kidney	Glomerular filtration rate/1.73 sq M.predicted [Volume Rate/Area] in Serum, Plasma or Blood	84	493	1.48e-02	Higher in HTN
metabolic	Serum potassium	55	78	1.51e-02	Lower in HTN
blood	Basophils [#/volume] in Blood	619	2551	1.56e-02	Higher in HTN
other	Oxygen saturation in Arterial blood	145	408	2.37e-02	Lower in HTN
kidney	Creatinine	1683	6246	2.44e-02	Higher in HTN
liver	Bilirubin.total	1413	5207	2.74e-02	Lower in HTN
other	Immature granulocytes [Presence] in Blood	84	447	3.12e-02	Higher in HTN
other	Sex hormone binding globulin	41	168	3.27e-02	Lower in HTN
kidney	Urea nitrogen	1744	6358	3.36e-02	Lower in HTN
blood	Variant lymphocytes/100 leukocytes in Blood	49	197	4.13e-02	Lower in HTN
other	Anion gap 4 in Serum or Plasma	35	73	4.26e-02	Higher in HTN
other	Epithelial cells.squamous [#/area] in Urine sediment	35	111	4.34e-02	Lower in HTN

159
